# Characterization of G-protein coupled receptors from the blackback land crab *Gecarcinus lateralis* Y organ transcriptome over the molt cycle

**DOI:** 10.1186/s12864-018-5363-9

**Published:** 2019-01-22

**Authors:** Nhut M. Tran, Donald L. Mykles, Abigail Elizur, Tomer Ventura

**Affiliations:** 1 GeneCology Research Centre, School of Science and Engineering University of the Sunshine Coast, Queensland, 4556 Australia; 2 Department of Biology, Colorado State University, Fort Collins, CO 80523 USA

**Keywords:** Neuroendocrine signalling pathways, Rhodopsin-like receptors, Secretin-like receptors, Decapod crustaceans, Ecdysis regulation, Crustacean Hyperglycemic Hormone family of neuropeptides, Molting, Ecdysteroids, G protein-coupled receptor

## Abstract

**Background:**

G-protein coupled receptors (GPCRs) are ancient, ubiquitous, constitute the largest family of transducing cell surface proteins, and are integral to cell communication via an array of ligands/neuropeptides. Molt inhibiting hormone (MIH) is a key neuropeptide that controls growth and reproduction in crustaceans by regulating the molt cycle. It inhibits ecdysone biosynthesis by a pair of endocrine glands (Y-organs; YOs) through binding a yet uncharacterized GPCR, which triggers a signalling cascade, leading to inhibition of the ecdysis sequence. When MIH release stops, ecdysone is synthesized and released to the hemolymph. A peak in ecdysone titer is followed by a molting event. A transcriptome of the blackback land crab *Gecarcinus lateralis* YOs across molt was utilized in this study to curate the list of GPCRs and their expression in order to better assess which GPCRs are involved in the molt process.

**Results:**

Ninety-nine *G. lateralis* putative GPCRs were obtained by screening the YO transcriptome against the Pfam database. Phylogenetic analysis classified 49 as class A (Rhodopsin-like receptor), 35 as class B (Secretin receptor), and 9 as class C (metabotropic glutamate). Further phylogenetic analysis of class A GPCRs identified neuropeptide GPCRs, including those for Allatostatin A, Allatostatin B, Bursicon, CCHamide, FMRFamide, Proctolin, Corazonin, Relaxin, and the biogenic amine Serotonin. Three GPCRs clustered with recently identified putative CHH receptors (CHHRs), and differential expression over the molt cycle suggests that they are associated with ecdysteroidogenesis regulation. Two putative Corazonin receptors showed much higher expression in the YOs compared with all other GPCRs, suggesting an important role in molt regulation.

**Conclusions:**

Molting requires an orchestrated regulation of YO ecdysteroid synthesis by multiple neuropeptides. In this study, we curated a comprehensive list of GPCRs expressed in the YO and followed their expression across the molt cycle. Three putative CHH receptors were identified and could include an MIH receptor whose activation negatively regulates molting. Orthologs of receptors that were found to be involved in molt regulation in insects were also identified, including LGR3 and Corazonin receptor, the latter of which was expressed at much higher level than all other receptors, suggesting a key role in YO regulation.

**Electronic supplementary material:**

The online version of this article (10.1186/s12864-018-5363-9) contains supplementary material, which is available to authorized users.

## Background

Crustaceans are a diverse subphylum of arthropods comprising of close to 67,000 species, classified into six classes, which together with their derived insects, comprise the pancrustacea superphylum clade [1]. Malacostraca is the most diverse and species rich class of crustaceans, including the decapod crustaceans, which comprise the more familiar groups of species, such as crabs, crayfishes, lobsters, and shrimps. Crustaceans are adapted to a wide range of conditions, which explains their wide distribution across many ecological niches. Apart from their ecological significance, decapod crustaceans are highly prized in fisheries and aquaculture. In 2015, nearly 7.4 billion tonnes of crustaceans were cultured worldwide, with nearly 90% of total production attributed to coastal regions of Asian countries [2]. Additionally, decapod crustaceans are listed among the worst invasive species globally [3–5]. This great importance necessitates a better understanding of key biological processes of crustaceans in order to address key bottlenecks in the fishery and aquaculture industries to enable it to meet the ever-growing demand in quality protein [6], as well as to devise species-specific treatments for invasive species. One potential application is monosex biotechnology to minimize the species’ invasive potential [7]. Growth control is another area of active research.

In crustaceans, like other arthropods, locomotion is facilitated by a rigid exoskeleton. Chitin, the second most abundant carbohydrate in nature following cellulose, is the major component of the exoskeleton, providing a scaffold for cuticular proteins that form links between the chitin fibers, as well as stabilize deposition of minerals that harden the cuticle. The skeletal muscles attach internally to the exoskeleton and contract to enable limb movements [8]. In some crustacean species, such as crabs, the rigid, highly calcified exoskeleton, provides protection against predators [9]. Unlike holometabolous insects (where following larval development there is a complete metamorphic transition through from pupae to adult, for example: flies, mosquitos etc.), crustaceans continually grow after they metamorphose into the juvenile stage. In order to grow and develop following metamorphosis, they must shed their old exoskeleton and produce a new larger one. In this process termed molting (or ecdysis) crustaceans synchronously form a new flexible exoskeleton under the existing one, absorb the minerals from the old exoskeleton, and then emerge from the old exoskeleton and harden the new one [10]. An advantage of continual growth is that crustaceans can increase in size and also regenerate lost limbs.

The molt cycle involves well-defined stages including intermolt, premolt, ecdysis, and postmolt. The longest stage is intermolt, when the animal accumulates organic compounds and stores the energy required for reproduction and molting. In premolt, the epidermal layer enlarges and starts to separate from the old exoskeleton. It then synthesizes the outer layers of the new exoskeleton, while degrading the inner layers of the old exoskeleton. Ecdysis then takes place, by which the animal emerges from its old exoskeleton. The postmolt stage begins by the rapid uptake of water and minerals (and in some species, minerals are also retrieved from internal storages that dynamically fill prior to ecdysis as pouches outside the stomach, called gastrolithes) and harden the new exoskeleton to complete the molt cycle. During this stage, the animals start to consume food [11]. Molting in arthropods is triggered by a peak in the hemolymph level of derivatives of the steroid hormone ecdysone, which is synthesized in a pair of endocrine glands, known as the Y-organs (YOs), located bilaterally in the cephalothorax. The YOs ecdysteroid production capacity varies over the molt cycle; it is highest in premolt and lowest in postmolt (see review [10]).

In decapod crustaceans, ecdysone is secreted from the YOs into the hemolymph and is carried to target tissues, where it is hydroxylated into the active molt hormone: 20-hydroxyecdysone (20HE) [12–16]. 20HE binds to the ecdysteroid receptor, which then initiates a signaling cascade of transcription factors that alter the molecular program for molting and metamorphosis [17–20]. The synthesis of ecdysteroids by the YO is inhibited by a neuropeptide known as the molt-inhibiting hormone (MIH), produced predominantly by the X-organ (XO) [21, 22]. The XOs are located bilaterally in the eyestalks and are responsible for producing a suite of endocrine factors conveyed to the nearby sinus gland (SG) for storage and regulated release to the hemolymph. These factors include MIH, crustacean hyperglycemic hormone (CHH), vitellogenesis-inhibiting hormone (VIH), mandibular organ-inhibiting hormone (MOIH), and ion transport peptide (ITP) [23]. From a practical point of view, most of the decapod crustaceans produced through aquaculture in farms globally require removal of the eyestalks of broodstock females in order to induce spawning in captivity [24–26]. A better understanding of the mechanism which regulates each process can lead to specific treatments that avoid the use of such an extreme and labor-intensive method.

Our model organism is arguably the most well studied decapod crustacean in the context of molting. One of the earliest scientific studies on *Gecarcinus lateralis* was published in 1952 by Bliss and Welsh [27], describing the XO-SG ultrastructure. Experimental studies by Skinner and later by Chang and Mykles have contributed to a better understanding of the hormonal regulation of molting [10, 11], as well as the development of tools to investigate the molting process. YO assays [28–30] and transcriptomics [31–34] have been used to investigate the signaling pathways controlling YO ecdysteroidogenesis. However, one mystery remains, which is the identity of the MIH receptor. Although CHH and MIH share a similar function of inhibiting ecdysteridogenesis by the YO, the signaling pathways of these two neuropeptides are distinct. A membrane guanylyl cyclase (GC-II) is considered as the receptor activated by CHH, resulting in immediate increase of the intracellular messenger guanosine 3', 5' cyclic monophosphate (cGMP) level. As proposed, an unidentified receptor activated by MIH temporarily increases the cAMP level followed by upregulation of cGMP (see [10, 35] for reviews). This suggests that the MIH receptor is not a GC-II. In 2009, Zmora *et al.* performed binding assays using radio-labeled MIH with YO membranes collected from blue crab juveniles in intermolt stage and hepatopancreas membrane of mature vitellogenic females. MIH was found to bind to both YO and hepatopancreas membranes, but with far higher affinity to the YO, suggesting that the main binding site of MIH is the YO membrane [36].

G-protein coupled receptors (GPCRs) are ancient, ubiquitous, constitute the largest gene family of transducing cell surface proteins, and are integral to cell communication [37–39]. All members of the GPCR gene family contain a domain of seven transmembrane α-helices with three extracellular loops and three intracellular loops [40]. The GPCR gene family is subdivided into three main classes depending on the pharmacological nature of their ligands and sequence similarity [41]. These are rhodopsin-like receptors (class A), secretin-like receptors (class B), and metabotropic-glutamate-receptor-like (class C), which represent about 89%, 7%, and 4%, respectively [42], of the known GPCRs [42]. In insects, most of the neuropeptide-activated receptors are predominantly rhodopsin-like receptors and some are secretin-like receptors [43]. GPCR sequences within these families can share less than 25% identity between species [44], making it difficult to annotate newly identified candidate receptors. More than 1,000 GPCRs have been characterized in *Caenorhabditis elegans* [45], while the number of GPCRs is over 200 in *Drosophila melanogaster* [46], adding another level of complexity presented by the vast variation of GPCR number across ecdysozoans. In crustaceans, there are many efforts to deorphanize neuropeptide receptors, but there are only a few species where a comprehensive list of GPCRs have been identified. Recently, advances in sequencing technologies have facilitated transcriptome-based annotation. In 2014, Nagai et al. characterized two GPCRs, BNGR-A2 and A34, as ITP receptors, and A24 as an ITP-like receptor (member of CHHR family) in the silkmonth *Bombyx mori*, using *in vitro* binding assays with ITP peptides and 30 GPCRs [47]. Using these ITP and ITP-like receptors from *B. mori* as references for phylogenetic study, Veenstra identified one contig (Pc-GPCRA9) as an ITP-like receptor based on clustering with BNGR-A24 and three receptors as putative ITP receptors (Pc-GPCRA52, Pc-GPCRA53, Pc-GPCRA63) in the red swamp crayfish *Procambarus clarkii,* based on clustering with BNGR-A34 [48]. A recent study in the eastern spiny lobster *Sagmariasus verreauxi* has also proposed two putative ITP receptors based on phylogenetic alignment to BNGR-A34 and Pc-GPCRA53 [49]. These studies, together with the high similarity shown between insect and decapod neuropeptidomes [50, 51], make it possible to predict the receptors for decapod neuropeptides based on those deorphanized in insects. This study curated a comprehensive list of GPCRs in the *G. lateralis* YO transcriptome across the molt cycle.

## Results and discussion

GPCRs play a central role in cell signaling as receptors for several transmitters, mediators, hormones, and neuropeptides. In crustaceans, most of the neuropeptides with known receptors act through GPCRs. Although many dozens of neuropeptides have been identified in hundreds of crustacean species over the last decade [48, 52, 53], information about their receptors (GPCRs) in terms of sequence, structure, and function, is very limited. Recent studies on decapod crustaceans formed a foundation for the future discovery of GPCRs. In 2015, Veenstra mined publically-available databases of *P. clarkii* and found several neuropeptide receptors, including those for Ast C, LGR, PDF, DH31, and DH44 [48]. In 2016, Buckley *et al.* computationally identified 86 GPCRs in the eastern spiny lobster *S. verreauxi*, including important neuropeptide receptors [49]. In this study, transcriptomic analysis identified 99 GPCRs in the *G. lateralis* YO. Of these, 71 were annotated either by phylogenetic or domain analysis, which include Ast receptor, Crz receptor, CHHamide receptor, and FMRFamide receptor. These outcomes are comparable to previous studies in Decapoda [48, 49]. N-glycosylation motif arrangement analysis of selected receptors was used to confirm the annotation.

*In silico* transcriptomic analysis identified 299 sequences with seven TM (Table 1). Ninety-nine of the 299 sequences, have the seven TM domain characteristic of GPCRs. Among them, 49 proteins were identified as Rhodopsin-like receptors (designated as GeclatGPCR_A1 to 45). Phylogenetic analysis and blast search using annotated GPCRs from other arthropods enabled annotation of 37 Rhodopsin-like receptors (class A GPCRs; Additional files 1, 2 and Table 2). Thirty-five GPCRs were classified as secretin-like (class B) GPCRs. Of the 35 class B receptors, one was annotated as diuretic hormone 44 (DH44) receptor, one diuretic hormone 31 (DH31) receptor, one parathyroid hormone (PTH) receptor, and three were annotated as putative pigment dispersing factor (PDF) receptors. Nine class C and six class F receptors were inferred based on BlastP results and the Frizzled domain in class F.

**Table 1:** The numbers of sequences comprising each of the Pfam accessions

Domain name	Pfam accession	Description of domain	Number of unique predicted peptide sequences
7tm_1	PF00001.16	7 transmembrane receptor (rhodopsin family)	108
7tm_2	PF00002.19	7 transmembrane receptor (Secretin family)	54
7tm_3	PF00003.17	7 transmembrane sweet-taste receptor of 3 GCPR	17
7tm_7	PF08395.7	7tm Chemosensory receptor	1
7TM_GPCR_Srsx	PF10320.4	Serpentine type 7TM GPCR chemoreceptor Srsx	31
7TM_GPCR_Srv	PF10323.4	Serpentine type 7TM GPCR chemoreceptor Srv	11
7TM_GPCR_Srw	PF10324.4	Serpentine type 7TM GPCR chemoreceptor Srw	15
7TM_GPCR_Srx	PF10328.4	Serpentine type 7TM GPCR chemoreceptor Srx	16
ABC_tran	PF00005.22	ABC transporter	159
ABC_tran_2	PF12848.2	ABC transporter	9
ABC_transp_aux	PF09822.4	ABC-type uncharacterized transport system	1
Frizzled	PF01534.12	Frizzled/Smoothened family membrane region	10
Na_Ca_ex	PF01699.19	Sodium/calcium exchanger protein	7
GpcrRhopsn4	PF10192.4	Rhodopsin-like GPCR transmembrane domain	4

**Table 2:** List of GPCR class A (Rhodopsin-like receptor)

ID	Predicted function	Predicted ligand
Gl_GPCR_A1	Allatostatin A receptor	Allatostatin A
Gl_GPCR_A2	TRH receptor	TRH
Gl_GPCR_A3	ETH receptor	ETH
Gl_GPCR_A4	GPA2/GPB5 receptor	GPA2/GPB5
Gl_GPCR_A4b	GPA2/GPB5 receptor	GPA2/GPB5
Gl_GPCR_A5	Allatostatin B receptor	Allatostatin B
Gl_GPCR_A5b	Allatostatin C receptor	Allatostatin C
Gl_GPCR_A6	Corazonin receptor	Corazonin
Gl_GPCR_A7	Corazonin receptor	Corazonin
Gl_GPCR_A8	CCHamide receptor	CCHamide
Gl_GPCR_A8b	CCHamide receptor	CCHamide
Gl_GPCR_A9	CHH-like receptor	CHH family
Gl_GPCR_A10	CHH-like receptor	CHH family
Gl_GPCR_A11	FMRFamide receptor	FMRFamide
Gl_GPCR_A12	CHH-like receptor	CHH family
Gl_GPCR_A13	Proctolin receptor	Proctolin
Gl_GPCR_A14	LGR-C1 receptor	Unknown
Gl_GPCR_A14b	LGR-C2 receptor	Unknown
Gl_GPCR_A15	Unknown receptor	Unknown
Gl_GPCR_A16	HPR1 receptor	Unknown
Gl_GPCR_A17	HPR1 receptor	Unknown
Gl_GPCR_A18	HPR1 receptor	Unknown
Gl_GPCR_A19	Bursicon receptor	Bursicon
Gl_GPCR_A20	Unknown receptor	Unknown
Gl_GPCR_A21	TIE receptor	Unknown
Gl_GPCR_A22	Unknown receptor	Unknown
Gl_GPCR_A23	Moody receptor	Unknown
Gl_GPCR_A24	Unknown receptor	Unknown
Gl_GPCR_A25	Unknown receptor	Unknown
Gl_GPCR_A26	sNPF receptor	sNPF
Gl_GPCR_A27	Unknown receptor	Unknown
Gl_GPCR_A28	Unknown receptor	Unknown
Gl_GPCR_A29	Unknown receptor	Unknown
Gl_GPCR_A30	Serotonin receptor	Serotonin
Gl_GPCR_A31	Unknown receptor	Unknown
Gl_GPCR_A32	Serotonin receptor	Serotonin
Gl_GPCR_A33	Adenosine receptor	Adenosine
Gl_GPCR_A34	Octopamine receptor	Octopamine
Gl_GPCR_A35	Prostaglandin receptor	Prostaglandin
Gl_GPCR_A36	Prostaglandin receptor	Prostaglandin
Gl_GPCR_A37	Prostaglandin receptor	Prostaglandin
Gl_GPCR_A38	Prostaglandin receptor	Prostaglandin
Gl_GPCR_A39	Peropsin receptor	Peropsin
Gl_GPCR_A40	FMRFamide receptor	FMRFamide
Gl_GPCR_A41	Unknown receptor	Unknown
Gl_GPCR_A42	Unknown receptor	Unknown
Gl_GPCR_A43	Unknown receptor	Unknown
Gl_GPCR_A44	HPR1 receptor	Unknown
Gl_GPCR_A45	CCAP receptor	CCAP

**Fig. 1:**
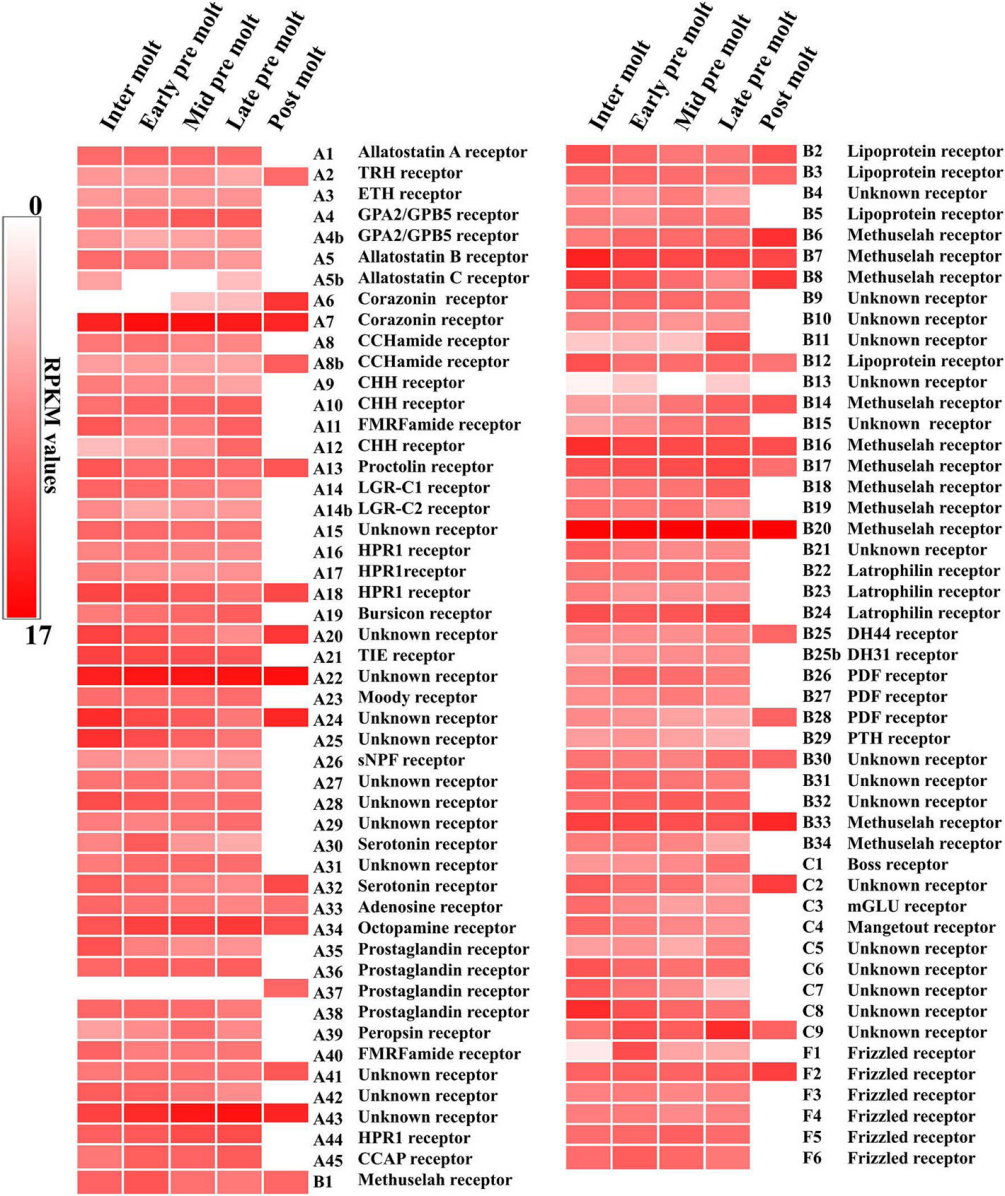
Gene expression (RPKM) heat map of GPCRs in different molt stages. Clustered by gene expression profile in a transcriptome dataset based on 5 different stages. Scores are coloured on a log2 scale with the red maximum and white minimum. Putative GPCR receptors are predicted based on a phylogenetic study and domain analysis

A heat-map profile of GPCR expression was generated based on reads mapping to the transcriptome database using a normalized read count (RPKM) [32] in five different molt stages. The RPKM of most GPCRs showed down-regulation at the postmolt stage (67% of the GPCRs; Fig. 1).

It is noteworthy that one GPCR (A37) showed specific expression at the postmolt stage, and one putative corazonin receptor (A6) was not expressed in the intermolt and early premolt stages, but was expressed in the mid and late premolt stages with higher expression in the postmolt stage. All three predicted CHHRs (A9, A10, A12) were expressed throughout the molt cycle except in the postmolt stage. GPCR families known to be involved with molting in arthropods and those that show differential expression in the present study are discussed in further detail below.

### Rhodopsin-like receptors (class A)

#### Allatostatin receptors

Allatostatins (ASTs) are pleiotropic neuropeptides that function as inhibitors of juvenile hormone (JH) production. JH is synthesized in the corpora allata in insects, while its crustacean active analogous compound (the JH precursor, methyl farnesoate; MF) is synthesized in the mandibular organ [54]. JH/MF maintains the appropriate stage and size and prevents metamorphosis [55]. Three types of ASTs have been identified in insects and characterized based on their conserved C-terminal sequences. The first class is FGLamide (Ast A), first discovered in cockroaches, which includes the conserved C-terminal sequence F-G-Lamide (other cases Y/F-X-F-G-L/Iamide) [56]. The second family of ASTs was isolated in crickets. These are C-terminally amidated peptides with tryptophan in the second and ninth positions, and are designated as the W(X)6Wamide or B-type ASTs [57, 58]. The third family of ASTs was first isolated in 1991 from the brain of the lepidopteran *Manduca sexta* [59]. It is a single 15 amino acid peptide with the nonamidated C-terminal pentapeptide P-I-S-C-F (Ast C). All three classes of peptides have since been identified in crustaceans [60–62].

**Fig. 2:**
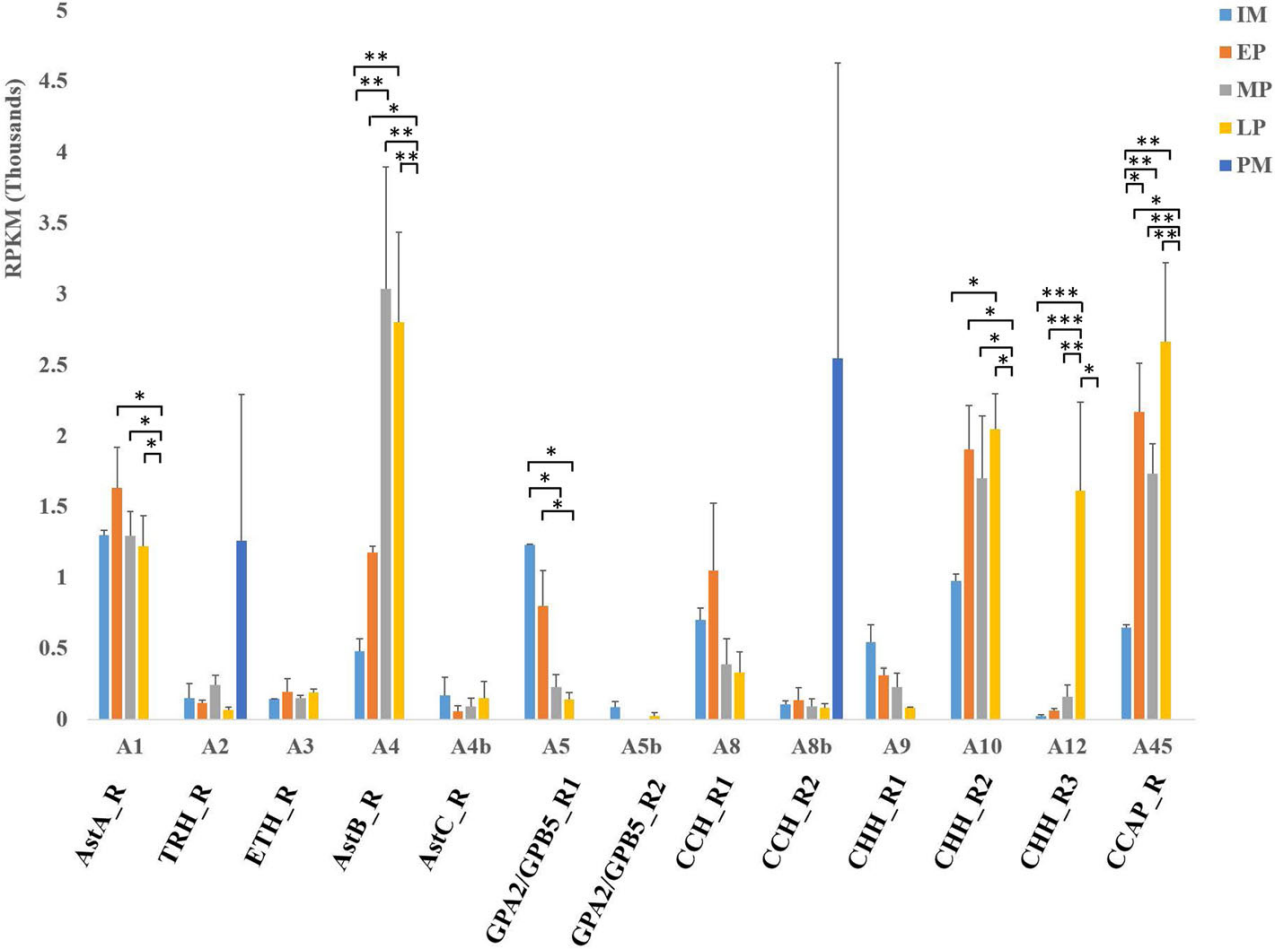
Statistical analysis of transcript RPKM expression. Molt-related GPCRs in *G. lateralis* YO were differentially expressed in five different stages of molt cycle (P < 0.05 and FDR < 0.05, highlighted in green in Additional file 2). Abbreviations: IM, intermolt stage; EP, early premolt stage; MP, mid premolt stage; LP, late premolt stage; and PM, postmolt stage. Significance level is marked as: * = *P* < 5E^-02^; ** = *P* < 5E^-04^; *** = *P* < 5E^-06^

Ast A regulates metabolism, feeding homeostasis, and energy mobilization by controlling release of glucagon-related adipokinetic hormone (AKH) and *Drosophila* insulin-like peptides (DILPs) [63]. One putative Ast A receptor (AstA_R; Gl-GPCRA1) was identified through phylogenetic analysis. *G. lateralis* Ast A receptor (AstA_R) contains three N-glycosylation motifs at the N-terminus and two N-glycosylation motifs at the C-terminus, while *P. clakii* AstA_R has three N-glycosylation motifs at the N-terminus and one at the C-terminus (Fig. 2a). In *G.lateralis*, the AstA_R was up-regulated during intermolt and premolt stages, and was not expressed at the postmolt stage, which is consistent with the role of Ast A in regulating metabolism and energy.

**Fig. 3:**
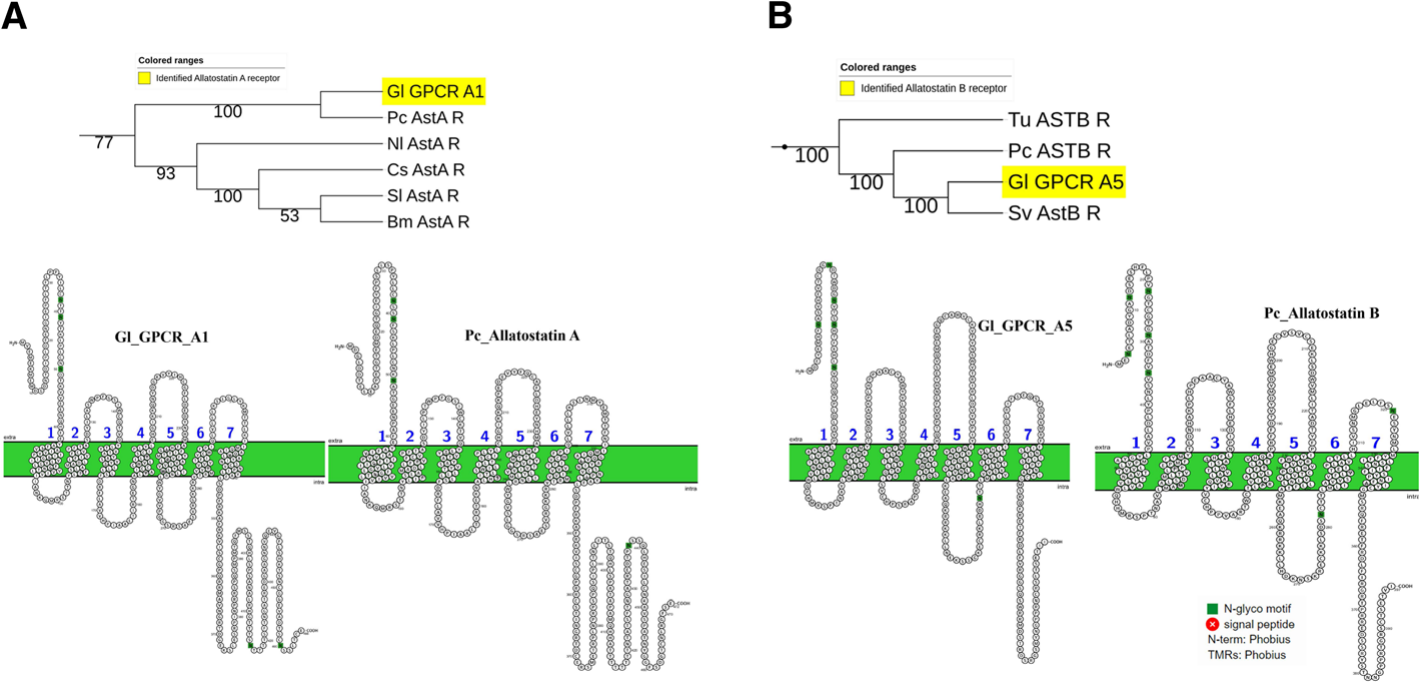
Pruned tree of Ast receptors and their amino acid sequence arrangement. **a** *G. lateralis* Ast A receptors in comparison with *P.clarkii* Ast A receptor and **b** *G. lateralis* Ast B receptors in comparison with *P. clarkii* Ast B receptor

Ast B is known for its myoinhibitory role and is therefore also referred to as myoinhibitory peptide (MIP) [64]. In *D. melanogaster*, Ast B (also called sex peptide in insects) blocks the receptivity of copulated females and increases food uptake after copulation [65]. Ast B is also known as a signaling molecule for settlement behavior in *Platynereis* larvae [66]*.* One putative Ast B receptor (AstB_R; Gl-GPCRA5) was identified, which clustered with AstB_R identified in *P. clarkii* (Fig. 2b). Gl-GPCRA5 showed the same RPKM expression trend as most other receptors, with the highest level in the intermolt stage and gradually decreasing in expression in premolt stages, with no expression at the postmolt stage (Fig. 3). This expression pattern is opposite to that found in holometabolous insects [11] (also based on expression data in *D. melanogaster* as found in Flybase; Sex peptide receptor (CG16752, FBgn0029768)), where AstB_R expression increases towards the molt and persists in the postmolt, when the animal is not feeding, suggesting a different role for Ast B in crustaceans. Another plausible explanation is that in this study we focused on the expression of AstB_R in the YOs, the crustacean analog of the prothoracic gland (PG) in insects, while the AstB_R temporal expression pattern in insects was examined in whole animals.

**Fig. 4:**
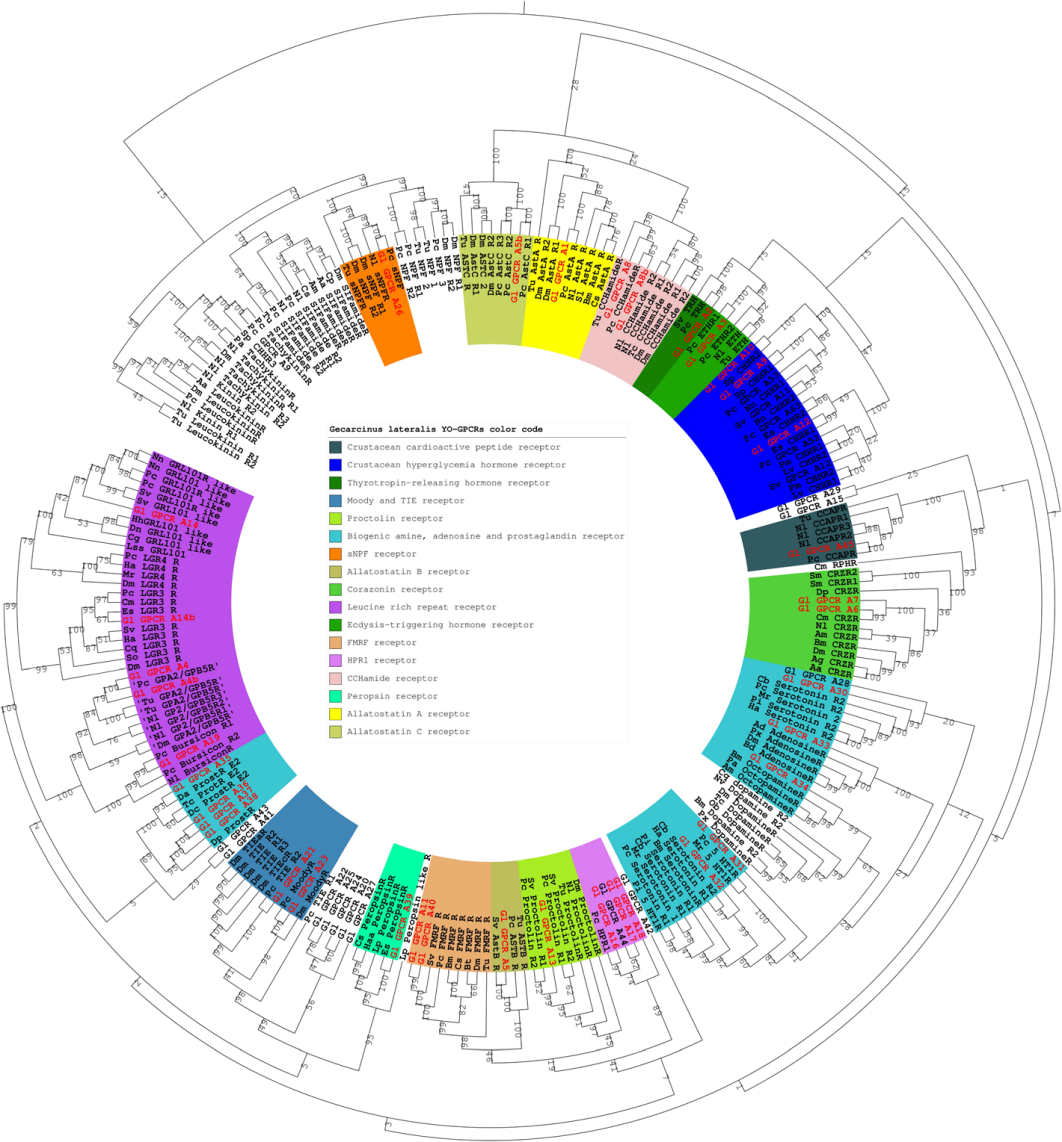
Phylogenetic tree of class A GPCRs presented as circular cladogram with different identified protein groups. The tree was constructed by the neighbor joining method with bootstap 1000 following multiple sequence alignment of 7TM regions in CLC workbench. Abbreviations: *Aa=Aedes aegypti, Ad = Anopheles darlingi, Ag = Anopheles gambiae, Am = Apis mellifera, Bd = Bactrocera dorsalis, Bm= Bombyx mori, Bt = Bombus terrestris, Cq = Culex quinquefasciatus, Cs= Callinectes sapidus, Dm= Drosophila melanogaster, Dp= Daphnia pulex, Es = Eriochier sinensis, Gl= Gecarcinus lateralis, Ha = Homarus americanus, Haa = Hasarius adansoni, Lp = Limulus polyphemus, Lv = Litopenaeus vannamei, Mr = Macrobrachium rosenbergii, Nl = Nilaparvata lugens, Nv = Nephrops norvegicus, Ob = Ooceraea biroi, Pa = Periplaneta americana, Pc= Procambarus clarkia, Pm = Penaeus monodon, Px = Plutella xylostella, Sm = Strigamia maritima, Sp = Scylla paramosain, Sv= Samariasus verreauxi, Tc= Tribolium castaneum, Tu= Tetranychus urticae*

Ast C or PISCF-type Ast was originally described as an unknown neuropeptide in *M. sexta* [67]. Its analog was then identified in several insect species, e.g. *D. melanogaster* [68], *Tribolium castaneum* [69], and later in the decapods *P. clarkii* [48] and *S. vereauxi* [49]. Ast C inhibits JH biosynthesis in *M. sexta*, *Helicoverpa zea* and *Aedes aegypti* [70]. It also functions as an immunosuppressive factor that prevents immunopathology or reduces unnecessary metabolic costs following microbial exposure [71]. One putative Ast C receptor (AstC_R; Gl_GPCRA5b) was predicted through phylogenetic analysis (Fig. 4). The RPKM expression of Gl-GPCRA5b remained at a low level throughout the molt cycle, and could not be detected at early premolt, mid premolt, and postmolt stages.

#### Corazonin receptors

Corazonin (Crz) was initially discovered as a strong cardioaccelerator in the American cockroach *Periplaneta americana* [72] and later in other insect species [73]. It is also found in decapod crustaceans [74–76]. Crz has various functions in different species. In locusts, Crz participates in the pigmentation process [77], while it is also recognized as a cardioaccelerator in the American cockroach [78]. In addition, Crz indirectly affects ecdysis in *M. sexta* [79], serving as an ecdysis initiator [80]. Although Crz receptors are well conserved in amino acid sequence across species, a number of isoforms have been discovered in insects, crustaceans, and ticks [78].

**Fig. 5:**
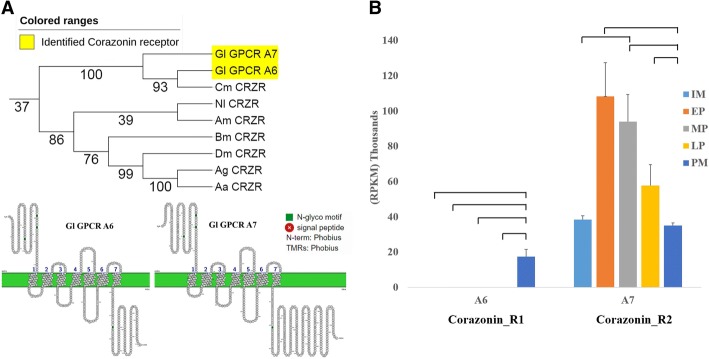
Pruned tree of Crz receptors and their amino acid sequence arrangement on their membrane*.* **a** *G. lateralis* Crz receptors and their sequence arrangement. **b** Statistical analysis of gene expression in term of RPKM for GPCRA6&7 in *G. lateralis* which were expressed in five different stages of molting cycle (P < 0.05 and FDR < 0.05, highlighted in red in Additional file 2)

Two putative Crz receptors were predicted in our study. Gl-GPCRA6 and Gl-GPCRA7 are clustered into the Crz receptor clade, sharing similar amino acid distribution in the seven TM domain with three N-glycosylation motifs at the N-terminus and one N-glycosylation motif at the C-terminus (Fig. 5a). Motif analysis indicates that both Crz receptors have identical amino acid sequences in the transmembrane domain, and a pairwise alignment shows high similarity in their sequences (72%). This suggests that both receptors are closely-related isoforms. GPCRA6 (Crz_R1) and A7 (Crz_R2) showed much higher expression in the YO (more than 10 times), compared with all other putative GPCRs. The expression pattern of these two putative receptors showed different trends. Crz_R1 showed no expression in the intermolt and early premolt stages, peaking at the postmolt stage. This expression suggests a role of Crz_R1 in postmolt. This is consistent with Alexander *et al*. (2017) who report that, in *C. maenas*, Crz_R1 (*C. maenas* clustered to Gl_GPCRA6 (Fig.. 5a)) is highly expressed in the YO, but it has little effect on ecdysteroid biosynthesis, except a modest stimulation in early postmolt [76]. While Crz_R1 peaked in expression in the postmolt, Crz_R2 showed high expression in intermolt, peaked in early premolt, and decreased towards the postmolt stage (Fig. 5b). Crz initiates ecdysis-triggering hormone (ETH) production in ‘inka’ cells in insects. ETH, in turn, triggers a signal cascade that leads to ecdysis. The elevated expression of the Crz receptors in the YOs reflects the key role of Crz receptors in molt regulation. Further studies are warranted to clarify the function of Crz_R2 in relation to the molt cycle in crustaceans.

#### CCHamide receptor

CCHamide is an invertebrate neuropeptide that was initially designated as ‘synthetic peptide, CCM’ [81]. Roller and colleagues assigned it with the new name CCHamide (CCHa) based on two conserved cysteines and an amidated histidine residue at the C terminus [82]. A comprehensive study found two CCHamide neuropeptides (CCHamide-1, CCHamide-2) in eleven insect species [53]. In *D. melanogaster,* CCHamide-2 is mainly located in endocrine cells in the gut, where the cells sense the quality of food and signal for the transport of CCHamide-2 to the brain, where it binds to a CCHamide-2 receptor and alters feeding behavior [83].

**Fig. 6:**
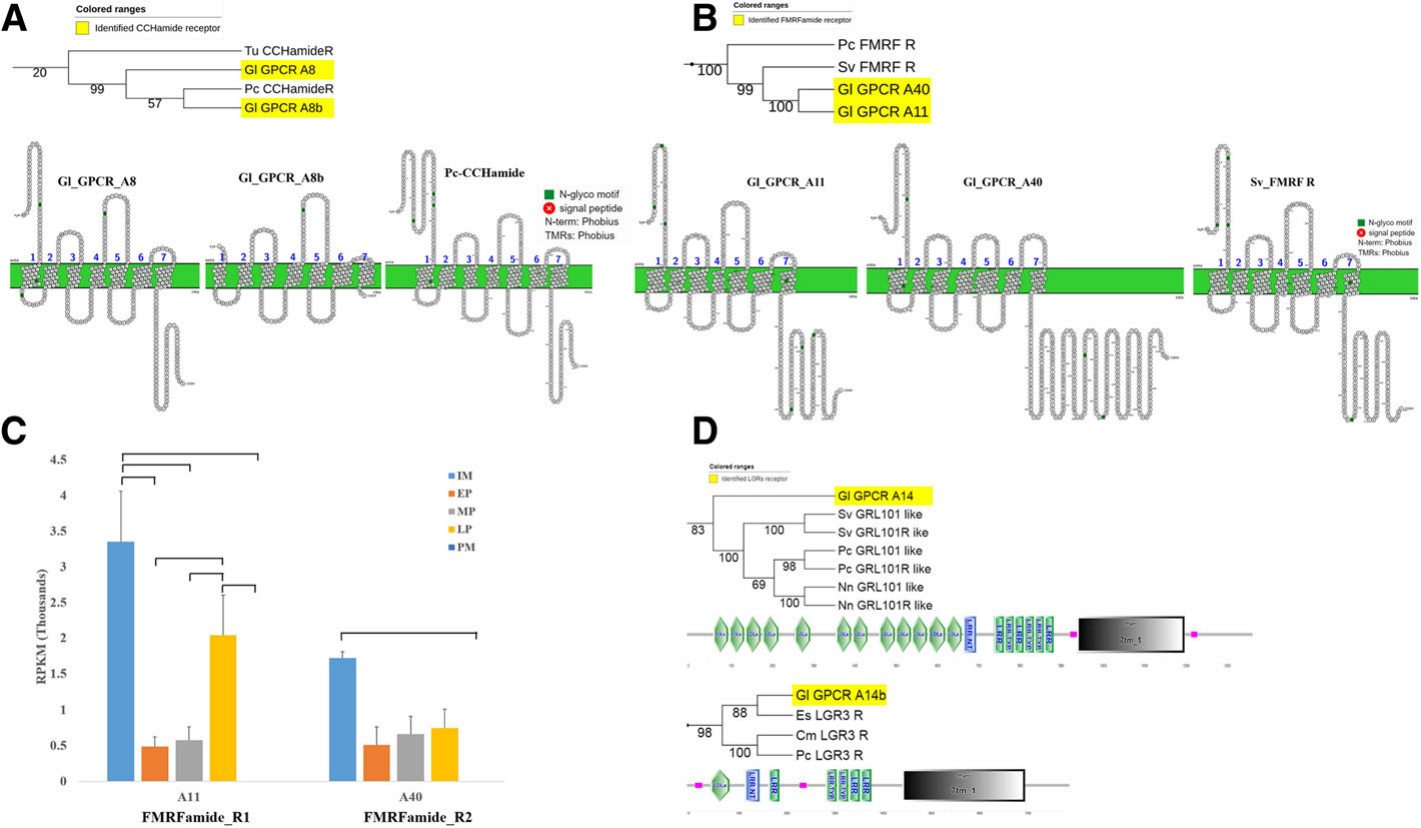
Putative CCHamide, FMRamide, GRL 101 like and LGR3 receptors. **a** & **b** Pruned tree of CHHamide and FMRFamide receptors and amino acid sequence arrangement of their putative receptors on their membrane. B) RPKM expression of FMRF receptor in five different molt stages. **c** RPKM expression of both putative FMRFamide receptor through molt stages. **d** LGR receptors with the number of LDLa motif, and LRR motif in the ectodomain. Abbreviations: IM, intermolt stage; EP, early premolt stage; MP, mid premolt stage; LP, late premolt stage; and PM, postmolt stage

Two putative CCHamide receptors were identified (CCH_R1; Gl-GPCRA8, and CHH_R2; Gl-GPCRA8b), which were clustered with *P. clarkii* CCHamide receptor in the phylogenetic analysis (Fig. 6a). CCH_R2 contained a partial sequence with an incomplete 7-TM domain. Comparison of the 7-TM domain distribution across the membrane between CCH_R1 and CCH_R2 showed one N-glycosylation motif in common at the second extracellular loop of both CCHRs. CCH_R1 showed high expression in all four molt stages except postmolt (Fig. 3). The rapid increase from the intermolt stage to early premolt stage, followed by a drop in expression at the mid and late premolt stages, and no expression at the postmolt stage also implicates CCHamide receptor in early premolt. CCH_R2 was expressed at low levels from the intermolt to late premolt stage, and peaked in expression at the postmolt stage (Fig. 3). The SEM of RPKM expression at postmolt stage of CCH_R2 was relatively high because of high variation between replicates. The great difference in RPKM expression between postmolt stage to other stages suggests an important role in the postmolt stage.

#### Crustacean cardioactive peptide receptors

Crustacean cardioactive peptide (CCAP) is produced in the pericardial organ of the shore crab *Carcinus maenas*, where it accelerates heart contraction [84]. It was later found in the pericardial organ of *Homarus americanus* and *Cancer productus* [85, 86]. CCAP also plays a role in stress response and biosynthesis adaptation in decapod crustaceans [87]. In *Macrobrachium nipponense*, CCAP is among six key neuropeptides found to be involved in reproduction regulation [88]. Although the specific name of CCAP applies to crustaceans, it also stimulates the heartbeat in insect species, such as *P. americana*, *D. melanogaster, Baculum extradentatum,* and *Locusta migratoria* [89]. CCAP also functions in ecdysis in several insect species. CCAP initiates the ecdysis motor program in *M. sexta* [90] and regulates timing of ecdysis behavior in *D. melanogater* [91]. One putative CCAP receptor (CCAP_R) was identified that clusters with *P. clarkii* CCAP_R in the phylogenetic tree (Fig. 4). RPKM expression analysis showed a similar expression to that of other receptors, with low expression at the intermolt stage and higher expression throughout the premolt stages, followed by no expression at the postmolt stage (Fig. 3).

#### CHH/CHH-like receptor

MIH is a neuropeptide that controls molting in decapod crustaceans. The identification of its receptor has been the focus of crustacean endocrinological research for decades. In 2014, Nagai *et al.* identified two putative GPCRs, BNGR-A2 and A34, as ITP receptors, and A24 as an ITP-like receptor (member of CHHR family) in the silk moth *B. mori*. This led to the identification of CHH-like family receptors in crustaceans. Veenstra identified three CHH-like receptors clustered with BNGR-34 in *P. clarkii*, and two putative CHH-like receptors have also been found in *S. verreauxi* [46].

**Fig. 7:**
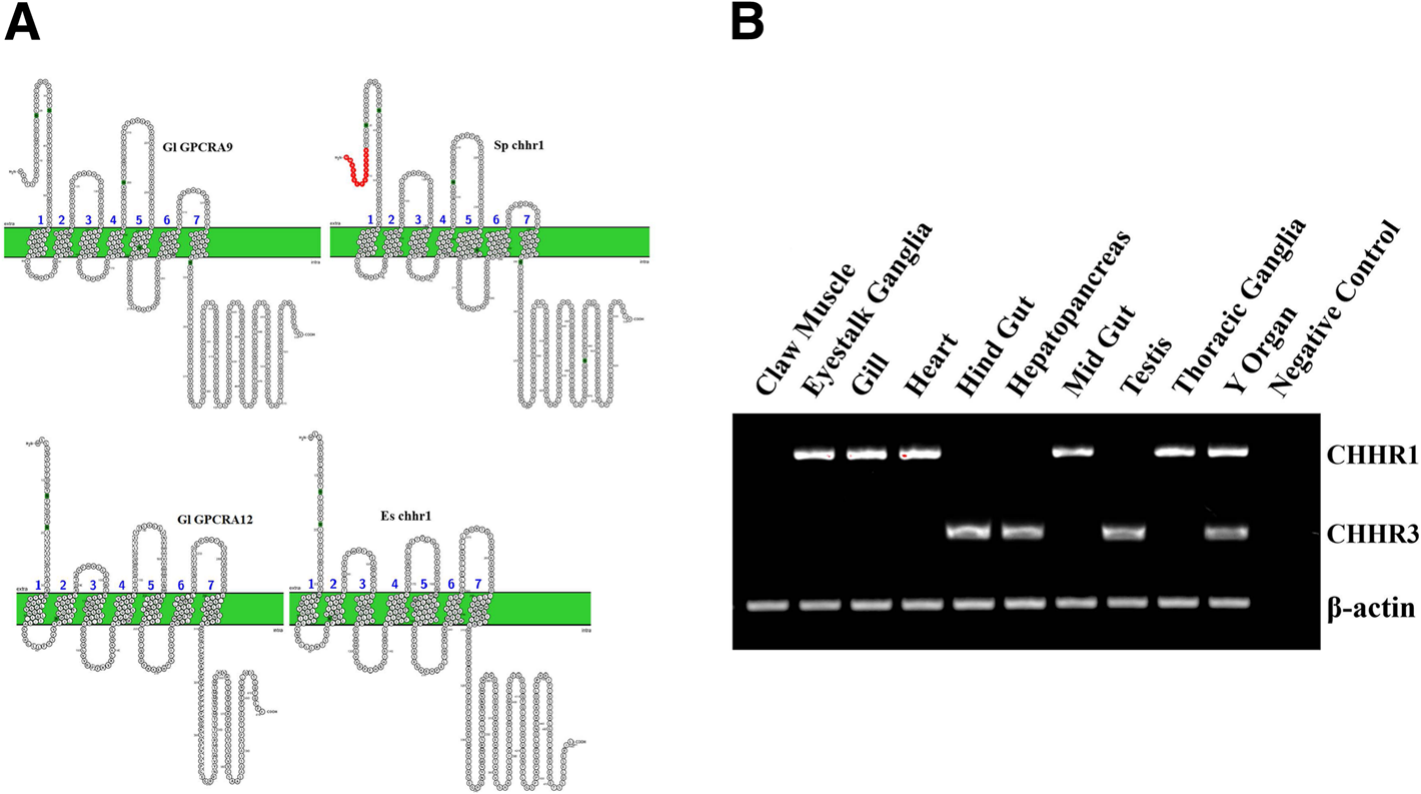
Putative CHH receptors and their tissue distribution. **a** Pruned tree of CHHRs and amino acid sequence arrangement of putative CHHRs. Transmembrane domains of both Gl_GPCRA9 and Gl_GPCRA12 were predicted using TMHMM online tool. **b** RT-PCR was carried out using cDNA from ten different organs of *G. lateralis*. Primers were designed to amplify two putative CHHR receptors (Gl_GPCRA9 and Gl_GPCRA12) (Table 1). Tissue expression pattern obtained from RT-PCR gel image visualized under UV light

Three putative GPCRs (Gl-GPCRA9, Gl-GPCRA10, Gl-GPCRA12) were identified as CHH-like receptors, as these sequences clustered into the CHHR clade (Fig. 7a). Further analysis of the transmembrane domains of these three proteins with TMHMM indicated that Gl-GPCRA9 and Gl-GPCRA10 contain a complete 7-TM domain, while Gl-GPCRA10 contains 6 transmembrane helices (data not shown). This could be explained by incomplete recovery of the sequence from the assembled contigs. Gl-GPCRA9 and Gl-GPCRA10 were expressed in all molt stages, while the expression of Gl-GPCRA12 decreased in late premolt and postmolt stages. Two CHHRs (Gl_GPCRA9 and Gl_GPCRA12) were examined using RT-PCR expressed in the YOs. Notably, both receptors were expressed not only in YOs, but they were also expressed in other tissues (Fig. 7b). Zmora *et al.* conducted MIH binding assays in the blue swimmer crab *Callinectes sapidus* [36]. This study showed that MIH bound predominantly to membranes extracted from the YOs but to a lesser extent also to membranes extracted from the hepatopancreas [36]. Based on this result, the spatial expression pattern of the putative CHHRs identified in this study cannot determine which receptor might be binding MIH and therefore receptor activation assays are required.

#### Ecdysis-triggering hormone receptors

Ecdysteroids are responsible for initiating the molting process, and are synthesised and released from the PGs in insects or from the YOs in crustaceans [10]. In insects, ecdysteroids trigger the production of ETH in ‘inka’ cells, a specialized group of endocrine cells scattered across the epithelial cells of the insect tracheae [92]. ETH production causes a surge in Eclosion Hormone (EH) secretion, which leads to the release of CCAP and upregulation of cGMP [93]. ETH travels to the central nervous system (CNS) where it stimulates the sensitivity of the CNS to ETH by promoting the expression of the ETH receptor (ETHR). In *M. sexta*, two alternatively spliced ETHRs (ETHR-A and ETHR-B) are encoded by *ethr* gene and expressed in discrete central neurons [94]. Previous studies indicated ETH is a key regulator that initiates ecdysis in insects [reviewed in [95]]. In *D. melanogater*, the *eth* gene encodes two neuropeptides designated ETH1 and ETH2. Injection of ETH1 into pharate pupae strongly induces proecdysis within 1-3 minutes, followed by ecdysis, while injection ETH2 has no effect [96]. One putative ETHR was identified and clustered with *P. clarkii* ETH_R1. ETHR was expressed at a similar level of RPKM through the molt cycle except the postmolt stage, where no expression was detected (Fig. 3).

#### FMRFamide/ FMRFamide-like receptor

FMRFamides are widely distributed neuropeptides with four signature amino acids at their C terminus (F-M-R-F) [97]. Many isoforms with variations of this signature tetrapeptide occur, which therefore classified as FMRFa-like peptides (FLPs) [98]. The first FLP was identified in *D. melanogaster* by cDNA cloning [99]. More FLPs have since been identified in insects using mass spectrometry [100, 101]. The FLP family comprises several neuropeptides that include sulfakinin, neuropeptide F (NPF), short neuropeptide F (sNPF), and myosuppressin [102–104]. FMRFamides are gut and heart contraction factors that also control digestive processes [105, 106]. Their role also extends to the ecdysis process by activating FMRFamide neurons during premolt in *D. melanogaster* [96] through direct innervation of the PG [107, 108].

Two *G. lateralis* putative FMRFamide receptors clustered with *P. clarkii* and *S. verreauxi* FMRFamide receptors (FMRF_R1; Gl_GPCRA11 and FMRF_R2; Gl_GPCRA40)*.* Motif analysis of the *S. serreauxi* and *G. lateralis* FMRFamide receptors showed high similarity between FMRF_R1 and FMRFamide receptor of *S. serreauxi*. In particular, there were three N-glycosylation motifs at the N-terminus and one N-glycosylation motif at the seven TM domain. The only difference was at the C-terminus where two additional N-glycosylation motifs were predicted in *G. lateralis* (Fig. 6b)*.* Motif analysis of FMRF_R2 showed distinct organization of one N-glycosylation motif at the N-terminus, two at the C-terminus, and one at the first TM domain. RPKM expression analysis showed a similar trend in both FMFFamide receptors, in which they were expressed high levels in the intermolt stage, slightly decreased in the next two molt stages, followed by an increase in the late premolt, and no expression in the postmolt stage (Fig. 6c). A small neuropeptide F (sNPF) receptor is also predicted based on the phylogenetic analysis (Gl-GPCRA26), where it clustered with the *P. clarkii* sNPF receptor (Fig. 4).

#### Leucine-rich repeats containing GPCRs

Leucine-rich repeats-containing GPCRs (LGRs) belong to the rhodopsin-like GPCR family. LGRs have, in addition to the GPCR-conserved 7-TM domain, multiple repeats of leucine-rich regions (LRRs) and low density lipoprotein (LDL) motifs for hormone binding. LGRs are classified into three types (A, B, and C) based on the number of LRRs, number of LDL motifs, and the structure of the hinge region [109]. Type A LGRs contain 7–9 LRRs and a long hinge region in their ectodomain, while type B LGRs typically have about twice the number of LRRs (16–18 LRR) and a shorter hinge region. In *D. melanogaster*, LGR1 (Type A LGR) is activated by GPA2/GPB5 neuropeptide, a heterodimer formed by GPA2 and GPB5 [110], and LGR2 (Type B LGR) is activated by bursicon [111]. Bursicon is a heterodimeric protein, consisting of α and β subunits. In *C. maenas*, Bursicon is co-localized with CCAP, both being released from neurons in the CNS. Bursicon plays a key role in cuticle hardening during post molt [112, 113]. Bursicon is also involved in reproduction by increasing vitellogenin and stimulating ovarian development in female shrimp, *Penaeus monodon* [114]. The number of LRRs in type C is similar to type A, but the hinge region is quite short and the LDL motifs are N-terminal to the LRRs. The Type C LGRs are subdivided into two subgroups: C1, which contains only one LDL motif and two cysteines in their hinge region and C2, which has five, six, ten or twelve LDLs N-terminal to the LRRs and four cysteines in their hinge region [109].

Five putative LGRs were predicted in the phylogenetic analysis. Gl_GPCRA4 and Gl_GPCRA19 clustered with the Bursicon receptor (type B LGR) and Gl_GPCRA4b clustered with the GP2/GP5 receptor (type A LGR). Another LGR identified (Gl-GPCRA14) clustered with GRL 101-like. Like other GRL 101-like receptors, Gl-GPCRA14 consists of 11 LDLs and 7 LRRs in its ectodomain, which defines it as type C2-like LGR (Fig. 6d). Gl-GPCR14b clustered into the LGR3 clade, belonging to type C1 LGR, as it contains 1 LDL and 6 LRRs in its ectodomain. In *D. melanogaster*, LGR3 is activated by Dilp8, a member of the insulin-like neuopeptide family [115]. LGR3 activation induces nitric oxide synthase production in the PG in response to Dilp8, which is elevated in injured imaginal discs [116, 117]. The increased nitric oxide synthase activity reduces ecdysone synthesis by the PG, which coordinates molting with the growth of the regenerating imaginal discs [118]*.* The identification of LGR3 in *G. lateralis* YOs suggests a similar function for this receptor in delaying the molt by damaged or lost limbs [11, 119, 120]. Autotomy of a regenerating limb in early premolt suspends molting until a secondary limb regenerate differentiates and grows to replace the lost regenerate (108). Secondary limb regenerates produce a peptide-like factor, designated limb autotomy factor - proedysis (LAF_pro_) (11), that delays molting by lowering hemolymph ecdysteroid titer (109). Given that several insulin-like peptides were recently identified in decapods [121, 122], it is possible that LAF_pro_ functions as the Dilp8 ortholog.

#### Thyrotropin-releasing hormone/ Thyrotropin-releasing hormone like receptors

In mammals, thyrotropin-releasing hormone (TRH) is a hypothalamic releasing factor that is synthesized mainly in the hypothalamus. Upon release TRH stimulates the release of thyroid-stimulating hormone and prolactin by the pituitary. TRH is also produced in peripheral tissues and the nervous system [123]. TRHs have a tripeptide Glu-His-Pro in their sequences and stimulate thyroid-stimulating hormone (TSH) biosynthesis [124]. In vertebrates, two TRH receptor (TRHR) isoforms are classified as type 1 (TRH-R1) and type 2 (TRH-R2). These two receptors belong to the rhodopsin/β-adrenergic receptor-like family of GPCRs and share up to 50% similarity in their amino acid sequences [125]. In arthropods, TRH-like receptors were identified both in insects (*Nilaparvata lugens, Rhodnius prolixus* [126]) and a crustacean *S. verreauxi* [49] by phylogenetic analysis. One putative *G. lateralis* TRHR clustered with *P. clarkii* and *S. verreauxi* TRHRs.

#### Biogenic amine, adenosine, and prostaglandin receptors

Biogenic amines are neuroactive molecules involved in synaptic transmission in the nervous system [127]. This group includes serotonin, dopamine, and octopamine. Serotonin (5-HT) increases blood glucose, while dopamine decreases blood glucose in hemolymph in several crustacean species [128]. Dopamine has a hyperglycemic effect in intact *P. clarkii* [129] and *Macrobrachium malcolmsonii* [130], but it has no effect on bilaterally eyestalk-ablated individuals. In insects, serotonergic neurons innervate the PG and control ecdysterodogenesis [131]. Autocrine signaling through the ββ3-octopamine receptor is essential for PTTH and insulin-like peptide stimulation of ecdysteroidogenesis in the *Drosophila* PG [132].

Three putative receptors clustered in the biogenic amine clade. Gl_GPCRA30 and Gl_GPCRA32 clustered with serotonin receptor 1 and serotonin receptor 2, respectively. Gl_GPCRA34 clustered with the octapamine receptor clade. One adenosine (Gl_GPCRA33) and four prostaglandin (Gl-GPCRA35-A38) receptors were also identified (Fig. 4). These results are comparable with previous studies in decapods [48, 49]. The expression of Gl_GPCR32-34 did not change significantly between stages in the YOs, while Gl_GPCR31, Gl_GPCR35-36, and Gl_GPCR38 show no expression in the postmolt stage.

#### Immune-related GPCRs

Crustaceans have an innate immune system to protect them from pathogenic bacteria and viruses. This immune system relies on the recognition of pathogen membrane proteins using pattern recognition proteins and defense using lectins, antimicrobial peptides, and clotting/melanization mechanisms [133]. A recent study on immune-related genes of *P. clarkii* activated by *Aeromonas hydrophila* infection identified a putative GPCR that is similar to HPR1 (protein receptor in hepatopancreas 1) [39]. Three putative receptors (Gl-GPCRA16, A17, A18, and A44) clustered with *P. clarkii* HPR1 in the phylogenetic analysis (Fig. 4). It is yet to be determined if they have a direct role in the innate immunity.

### Secretin-like family

#### Class B1

**Fig. 8:**
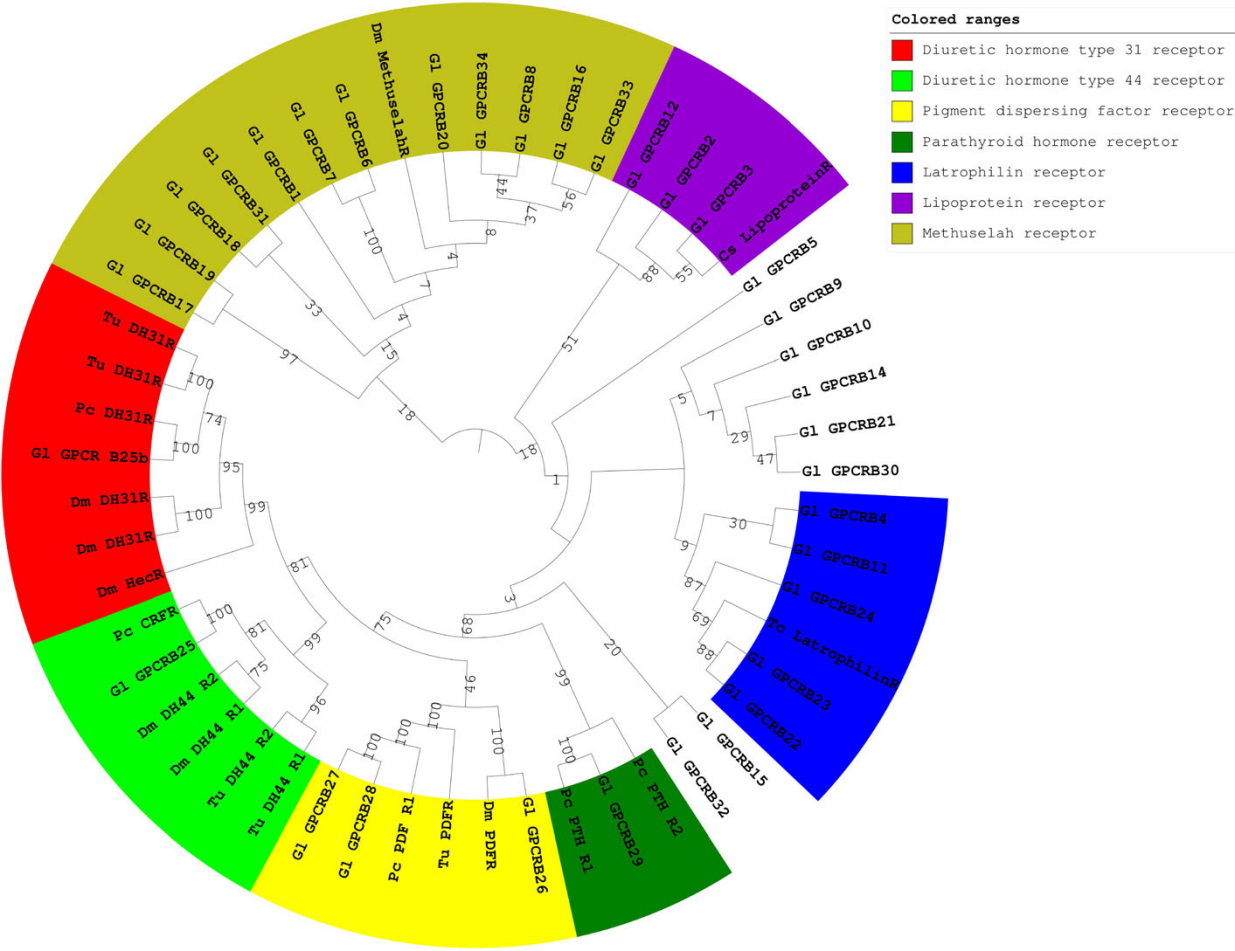
Phylogenetic tree of class B GPCRs presented as circular cladogram. Five protein groups were identified, including latrophilin, lipoprotein, methuselah, PDF, and DH44 receptor. The phylogenetic trees were constructed by neighbor joining method with bootstrap 1000 following multiple sequence alignment of 7-TM regions in CLC workbench. Abbreviations: *Cs= Callinectes sapidus, Dm= Drosophila melanogaster, Dp= Daphnia pulex, Gl= Gecarcinus lateralis, Pc= Procambarus clarkii, Tc= Tribolium castaneum, Tu= Tetranychus urticae*

Diuretic hormones (DH) regulate water balance in arthropods [134]. There are three primary insect DHs: corticotropin-releasing factor (CRF)-related peptides, calcitonin (CT)-like peptides, and the insect kinins [135]. CRF is structurally related to mammalian corticotropin, and is called Drome-DH31 in *D. melanogaster* [136]. DH31 was recently identified in several tissues of the green shore crab *C. maenas* and its function in rhythmic coordination was established [137]. CT peptide is structurally related to mammalian calcitonin-like peptide and is called Drome-DH44 in *D. melanogaster* [138]. One putative diuretic hormone type 44 (DH44) receptor (Gl-GPCRB25) and one diuretic hormone type 31 (DH31) receptor (Gl-GPCRB25b), were identified by phylogenetic analysis (Fig. 8).

Pigment dispersing factor (PDF) is a neuropeptide produced by the XO in crustaceans, and is found across inverterates. It has a variety of functions, including pigment dispersal in chromatophore cells [139] and regulation of locomotion behavior and egg-laying [140]. In *B. mori*, PDF binds to the BNGR-B2 receptor and stimulates the ecdysone biosynthesis in the PGs [141]. Decapod PDF receptors have been identified based on similarity with a gene from *D. melanogaster* (CG13758), including those in *P. clarkii* and *M. rosenbergii* [48, 142]. Three putative PDF/PDF-like receptors were identified in our study (Gl-GPCRB26-A28).

Parathyroid hormone (PTH) belongs to the parathyroid hormone family, which includes PTH, PTH-related peptide (PTHrP), and tuberoinfundibular peptide (PTH2). In vertebrates, the PTH family regulates the calcium titer in serum, affecting most organs [143]. One putative PTH receptor (PTHR) in the *G. lateralis* YOs transcriptome clustered with *P. clarkii* PTHR in the phylogenetic tree (Fig. 8).

#### Class B2

The class B2 of the secretin-like receptor family includes the calcium-independent receptor, brain-specific angiogenesis inhibitor, starry night receptor, latrophilin receptor, HE6 receptor, and homologs of the vertebrate adhesion receptor [144]. Class B2 members share a structural similarity in the form of a long extracellular N-terminus in the ectodomain, which consists of cleavage and binding sites, such as proteolytic site (GPS) and epidermal growth factor (EGF) domains [145]. Five putative latrophilin receptors (Gl-GPCRB4, B11, B22-24) and two lipoprotein receptors (Gl-GPCRB2, B3 and B12) were identified in the *G. lateralis* YO transcriptome (Fig. 8). The expression did not change significantly between stages in the YOs.

#### Class B3 (Methuselah-type receptors)

Methuselah/Methuselah-like was originally identified in *D. melanogaster* [146], and named after the methuselah gene. Most methuselah receptors contain conserved cysteine residues and glycosylation sites [147, 148]. This subfamily comprises 15 paralogs based on the similarity in their ectodomain, as well as a 7-TM domain [149]. Methuselah/Methuselah-like are found in several insects, such as *T. castaneum* and *B. mori* [150, 151]. They are involved in stress response and are associated with extended lifespan in *D. melanogaster* [146]. Mth2 in *T. castaneum* also plays a role in heat resistance and eclosion [152]. Twelve variants of the putative methuselah (Mth) receptor in *G. lateralis* clustered with *D. melanogaster* Mth receptor (Gl-GPCRB1, B6-8, B17-20, B31, and B33-34) (Fig. 8).

#### Class C

**Fig. 9:**
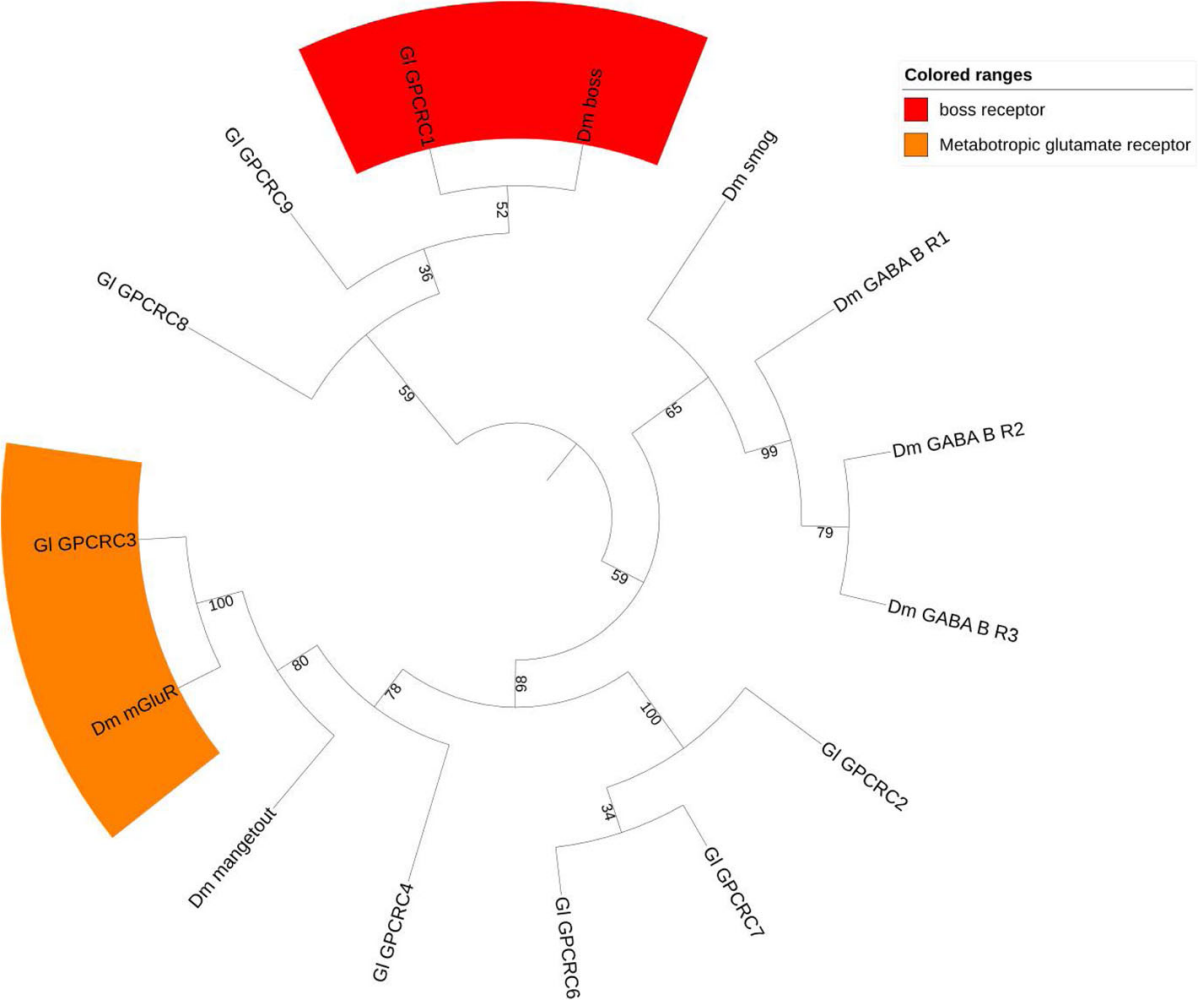
Phylogenetic tree of class C GPCRs presented as circular cladogram. Two protein groups were identified, including mGlu receptor and boss receptor. The phylogenetic trees were constructed by neighbor joining method with bootstrap 1000 following multiple sequence alignment of 7-TM regions in CLC workbench. Abbreviations: *Cs= Callinectes sapidus, Dm= Drosophila melanogaster, Dp= Daphnia pulex, Gl= Gecarcinus lateralis, Pc= Procambarus clarkii, Tc= Tribolium castaneum, Tu= Tetranychus urticae*

Class C GPCRs are classified based on their sequence phylogeny and conservation in the 7-TM domain [114]. These receptors possess a large (hundreds of residues) N-terminal extracellular domain [115]. Class C GPCRs include metabotropic glutamate (mGlu), γ-aminobutyric acid (GABA), Ca^2+^-sensing (CaS), sweet and amino acid taste, pheromone, and odorant receptors in fish, as well as several orphan receptors [114]. One putative mGlu receptor (Gl-GPCRC3) and one putative boss receptor (Gl-GPCRC1) were identified, as they clustered with *D. melanogaster* mGlu and boss receptors, respectively (Fig. 9).

#### Notable ommisions

Several GPCRs that are conserved across arthropods were not identified in the *G. lateralis* YO transcriptome, perhaps due to high divergence of these GPCRs between crustaceans and insects, or simply because they are not expressed in the YO. These include NPF, CNMamide, and Tachykinin receptors. A Blastp search against nr database at NCBI showed Gl_GPCRA24 as an NPF receptor but with low identity (maximum of 23%) and marginal E-value (higher than 1.0E^-7^). With the rapid expanding availability of transcriptomes and genomes of decapod species, the spatial-temporal expression pattern and genomic context of these receptors is expected to be elucidated.

## Conclusions

Bioinformatic analysis of RNA-Seq data provides a comprehensive and cost-effective method to catalog and annotate sequences and discover novel sequences (31). Using this approach, we identified contigs encoding 99 GPCRs in the YO transcriptome of *G. lateralis* (Fig. 1). The GPCRs were distributed between the three known GPCR classes. Seventy-two contigs were assigned to 17 identified GCPR groups based on ligand binding specificity and structural motifs (Fig. 4); 27 (27%) remained unidentified (Fig. 1). These data suggest that the YO has at least the potential for responding to a large number and diversity of ligands. Moreover, most were differentially expressed over the molt cycle (Figs. 1, 2, 5, and 6), suggesting that the sensitivity of the YO to these ligands is molt stage-specific. A striking example is the gene expression pattern of the repressed YO in postmolt animals. The repressed YO is characterized by low global gene expression (33). It is hypothesized that this low transcriptional activity prevents YO activation until the synthesis of the new exoskeleton is completed and the animal is in the intermolt stage (33). The majority (67%) of the GPCR contigs follow this pattern of higher levels in intermolt and premolt stages and low levels in postmolt (Fig. 1). However, 33 contigs were expressed at high or their highest levels in postmolt and one in particular, a progesterone receptor (A37), was only expressed in postmolt (Fig. 1), suggesting that these genes are involved in maintaining the repressed state and/or are involved in preparing the YO to transition to the basal state in the intermolt stage (33).

The analysis identified several GPCRs expressed in the YO that are of special interest. The proposed model for MIH signaling pathway consists of a transient cAMP/Ca^2+^-dependent triggering phase and a prolonged NO/cGMP-dependent summation phase, which inhibits YO ecdysteroid secretion between MIH pulses (10, 14, 28). The identity of the MIH receptor has remained elusive, but it is assumed that it is a GPCR, the activation of which initates the triggering phase. Three contigs, designated CHH_1 (A9), CHH_2 (A10), and CHH_3 (A12), were identified as receptors for the CHH neuropeptide family, which includes MIH, CHH, and ILP (13, 14, 22), and therefore are candidates for the MIH receptor (Figs. 1, 7). CHH_2 was expressed at high levels in the YOs from intermolt and premolt animals (Fig. 2), making it the leading candidate. However, conclusive identification of the MIH receptor awaits a functional assay that shows MIH activation of MIH receptor candidates expressed in a heterologous reporting system [15, 153]. The identification of FMRFamide, serotonin, and octopamine receptors raise the possibility that the YO is controlled by direct innervation. To our knowledge, innervation of the YO has yet to be demonstrated. However, the insect PG receives neuronal projections that directly control ecdysteroidogenesis [154]. FMRFamides and FMRFamide-related peptides bind to the myosuppressin receptor and inhibit ecdysteroidogenesis by lowering cAMP production [107, 108]. Serotonergic neurons innervating the *Drosophila* PG stimulate ecdysteroidogenic activity [131]. GABA and dopamine have indirect effects on the *P. americana* PG, as they have an inhibitory and excitory effect, respectively, on the activity of the PG nerve [155]. The identification of an octopamine receptor suggests that octopamine has a direct effect on the YO. However, to our knowledge, there are no published studies determining the effects of octopamine on YO ecdysteroid secretion. A Crz receptor was identified in the YO transcriptome that may be involved with molt regulation (Fig. 5) [76]. Finally, LGR3 may provide a parallel pathway in decapods and dipterans for the coordination of molting and tissue regeneration. LGR3 in the YO may mediate the suspension of molt by LAF_pro_ that is released by secondary limb regenerates, much like Dilp8, released from damaged imaginal discs, inhibits the PG. Transcriptomics has revealed that neuropeptide control of the YO is becoming more complex.

## Methods

### Transcriptome analysis

The transcriptome as well as FASTQ of *G. lateralis* YOs were obtained from a previous study [34]. In brief, the animals were collected from the Dominican Republic, shipped by air to Colorado, and maintained as described [29]. Animals were immobilized by severing the brain before removing sections of the carapace containing the YO. Animals were frozen at -20 ˚C. The animals were induced to molt using multiple leg autotomy (8 walking legs) and the YOs from 2-3 individuals were then collected from animals at the same molt stage. Five different molt stages including intermolt (IM - stage C_4_), early premolt (EP - stage D_0_), mid premolt (MP - stage D_1_), late premolt (LP - stage D_2_) and postmolt (PM), were collected and sequenced in triplicates (a total of 15 libraries). The transcriptomic data was screened for the longest open reading frames (ORFs) using transdecoder (version 5.0), generating a fasta file with the amino acid sequences. The amino acid sequences were scanned against the PfamA database (version 27.0) using hidden Markov models (HMMs) to identify the seven TM families. Sequences with seven TM profile were extracted (represented in Table 1). Transmembrane HMM (TMHMM) scan was then applied in parallel on the *G. lateralis* YOs ORF file to find the predicted helices. Two lists of PFAM HMM and TMHMM search outputs were cross-referenced to remove duplicates. The seven TM sequences were then categorized into GPCR subclasses comprising rhodopsin-like (class A), secretin and adhesion (class B), metabotropic glutamate (class C) based on Pfam. The references list were created using annotated sequences from previous studies [49, 156], and the BLAST analysis of GPCR protein sequences from YO against protein sequences from the NCBI non-redundant (nr) database with *Arthropoda* organism filter, and protein sequences blasted against the FlyBase database. The reference list was entered into TMMHMM [157] using default parameters to obtain the seven TM domain of each protein sequence.

The Reads Per Kilobase of transcript, per Million mapped reads (RPKM) were obtained by mapping sequence reads against YOs database using the RNA-seq tool of CLC Genomics Workbench (CLC Bio, version 10.0) with default parameters. The RPKM was calculated as follows:

$$ \mathrm{RPKM}=\frac{\mathrm{Total}\ \mathrm{exon}\ \mathrm{reads}}{\mathrm{mapped}\ \mathrm{reads}\left(\mathrm{millions}\right)\mathrm{x}\ \mathrm{exon}\ \mathrm{length}\ \left(\mathrm{KB}\right)} $$

The RPKM values of proteins relating to molt cycle were then imported into CLC for statistical analysis to determine whether there were significant differences in RPKM between different molt stages. The Empirical analysis of DGE was performed to compare RPKM between molt stages with the probability distribution less than 5% (*P* < 0.05). To avoid false positive result, the P values were then corected using false discovery rate (FDR < 0.05).

### Phylogenetic and functional study of GPCR families

To further annotate the *G. lateralis* GPCRs for phylogenetic analysis, the seven TM domains of all GPCRs were extracted and compiled with the reference list. Multiple sequence alignment was carried out using MUSCLE tools implemented in CLC Genomics Workbench (CLC Bio, version 10.0). The sequence alignment file was used to generate a phylogenetic tree with CLC Genomics Workbench (Neighbor-joining phylogeny with 1,000 bootstraps). The lists of GPCRs used for phylogeny are given in Additional file 1.

### Tissue specific expression of predicted CHHR by RT-PCR

Tissues were harvested from three adult intermolt *G. lateralis* males. A competitive ELISA assay was performed to confirm that the animals were in intermolt (stage C_4_) by measuring hemolymph ecdysteroid titer (19.0 ± 2.3 pg/μμl, n = 7). The samples were stored in RNA later at -20 °C until extraction. Total RNA was extracted from 10 tissues comprising claw muscle (CM), eyestalk ganglia (ESG), gill (G), heart (H), hindgut (HG), hepatopancreas (HP), midgut (MG), testis (T), thoracic ganglia (TG), and Y-organs (YOs). Trizol® Reagent (Invitrogen), was used to extract total RNA according to manufacturer’s instruction and quantified using a ND-2000 (NanoDrop Technologies, DE, U.S). The extractions were stored at -80 °C for RT-PCR experiment. Primers were designed using Primer 3 (http://bioinfo.ut.ee/primer3-0.4.0/) and synthesized by Sigma–Aldrich company (Table 3). The stored RNA was then converted into cDNA by reverse-transcription reaction in which 1 μμg RNA of each sample was used as templates, using Tetro cDNA synthesis kit (Bioline, Australia) following manufacturer’s instructions. PCR was performed using Mytaq Red kit (Bioline, Australia), complemented with 1 μμl of cDNA as the template, 0.8 nM of forward and reverse primers and up to 20 μμl DNase-free water. Touchdown PCR was set up as follows: 94 °C for 3 min, followed by 5 cycles of 94 °C for 30 s, annealing at 62 °C for 30 s and gradually decrease 1 °C in each cycle, elongation at 72 °C for 45s. Another 35 cycles included denaturation at 94 °C for 30 s, annealing at 55 °C for 30 s, elongation at 72 °C for 45 s, followed by a final extension at 72 °C for 10 min. The amplicons were then loaded on 1 % agarose gel stained with ethidium bromide, in TBE buffer and electrophorased at 90 volts for 60 min and visualized under UV light.

**Table 3:** Set of primers used for RT-PCR

Identifer	Forward primers	Reverse primers
Gl-GPCRA9	ggccaacagagtgattgcaa	gtgacgtcggtggtagatcc
Gl-GPCRA12	tgactacaacttcacggacag agc	gtacacgtgcatcttgttcacctc
Beta-actin	ctgacaccactccaccatgt	tcatagatggggacggtgtg

## Additional files

Additional file 1: **Table S1.** List of curated GPCRs using phylogenetic study and Blast search analysis. GPCRs subgroup clasification was defined using Pfam database and reference list. (XLSX 159 kb)

Additional file 2: **Table S2.** Statistical analysis of defined GPCRs using empirical analysis of DGE and false discovery rate method. (XLSX 80 kb)

## Abbreviations

20HE: 20-hydroxyecdysone
20HE: 20-hydroxyecdysone
CHHRs: Crustacean hyperglycemic hormone receptors
CHHRs: Crustacean hyperglycemic hormone receptors
GC-II: Guanylyl cyclase II
GC-II: Guanylyl cyclase II
GPCRs: G-protein coupled receptors
ITP: Ion transport peptide
ITP: Ion transport peptide
MIH: Molt inhibiting hormone
MIH: Molt inhibiting hormone
MOIH: Mandibular organ-inhibiting hormone
MOIH: Mandibular organ-inhibiting hormone
SG: Sinus gland
SG: Sinus gland
VIH: Vitellogenesis-inhibiting hormone
VIH: Vitellogenesis-inhibiting hormone
XO: X-organ
XO: X-organ
YOs: Y-organs
YOs: Y-organsGPCRs: G-protein coupled receptors

## References

[1] Von Reumont BM, Jenner RA, Wills MA, Dell'ampio E, Pass G, Ebersberger I, Meyer B, Koenemann S, Iliffe TM, Stamatakis A (2012). Pancrustacean phylogeny in the light of new phylogenomic data: support for Remipedia as the possible sister group of Hexapoda. Mol Biol Evol.

[2] Zhou X (2015). An overview of recently published global aquaculture statistics. FAO Fisheries and Aquaculture.

[3] Holdich DM, Pöckl M. Invasive crustaceans in European inland waters. In: Gherardi F. (eds) Biological invaders in inland waters: Profiles, distribution, and threats. Invading Nature - Springer Series In Invasion Ecology. Dordrecht: Springer; 2007;2.

[4] Gherardi F. (2007) Understanding the impact of invasive crayfish. In: Gherardi F. (eds) Biological invaders in inland waters: Profiles, distribution, and threats. Invading Nature - Springer Series In Invasion Ecology. Dordrecht: Springer; 2007;2.

[5] Gherardi F. Measuring the impact of freshwater NIS: what are we missing?. In: Gherardi F. (eds) Biological invaders in inland waters: Profiles, distribution, and threats. Invading Nature - Springer Series In Invasion Ecology. Dordrecht: Springer; 2007;2.

[6] Grealis E, Hynes S, O’Donoghue C, Vega A, Van Osch S, Twomey C (2017). The economic impact of aquaculture expansion: an input-output approach. Mar Policy.

[7] Ventura T, Sagi A (2012). The insulin-like androgenic gland hormone in crustaceans: from a single gene silencing to a wide array of sexual manipulation-based biotechnologies. Biotechnol Adv.

[8] Znidarsic N, Mrak P, Tusek-Znidaric M, Strus J (2012). Exoskeleton anchoring to tendon cells and muscles in molting isopod crustaceans. Zookeys.

[9] Moret Y, Jérôme M (2012). The immune role of the arthropod exoskeleton. ISJ-Invert Surviv J.

[10] Chang ES, Mykles DL (2011). Regulation of crustacean molting: a review and our perspectives. Gen Comp Endocrinol.

[11] Skinner DM (1985). Moulting and Regeneration. *The Biology of Crustacea - Integument, Pigments, and Hormonal Processes*.

[12] Riddiford LM, Hiruma K, Zhou X, Nelson CA (2003). Insights into the molecular basis of the hormonal control of molting and metamorphosis from *Manduca sexta* and *Drosophila melanogaster*. Insect Biochem Molec.

[13] Webster SG, Keller R, Dircksen H (2012). The CHH-superfamily of multifunctional peptide hormones controlling crustacean metabolism, osmoregulation, moulting, and reproduction. Gen Comp Endocrinol.

[14] Webster SG, Chang ES, Thiel M (2015). Endocrinology of molting. *The Natural History of Crustacea: Physiology*.

[15] Ventura T, Bose U, Fitzgibbon QP, Smith GG, Shaw PN, Cummins SF, Elizur A (2017). CYP450s analysis across spiny lobster metamorphosis identifies a long sought missing link in crustacean development. J Steroid Biochem Mol Biol.

[16] Mykles DL (2011). Ecdysteroid metabolism in crustaceans. J Steroid Biochem Mol Biol.

[17] Li T-R, White KP (2003). Tissue-specific gene expression and ecdysone-regulated genomic networks in *Drosophila*. Developmental Cell.

[18] Fahrbach SE, Smagghe G, Velarde RA (2012). Insect nuclear receptors. Annu Rev Entomol.

[19] Riddiford LM, Cherbas P, Truman JW. Ecdysone receptors and their biological actions. Vitamins Hormones. 2000;60:1–73. 10.1016/s0083-6729(00)60016-x 11037621

[20] Ventura T, Palero F, Rotllant G and Fitzgibbon QP. Crustacean metamorphosis: an omics perspective. Hydrobiologia. 2017;825(1):47–60.

[21] Hopkins PM (2012). The eyes have it: A brief history of crustacean neuroendocrinology. Gen Comp Endocrinol.

[22] Spaziani E, Wang WL (1993). Biosynthesis of ecdysteroid hormones by crustacean Y-organs: conversion of cholesterol to 7-dehydrocholesterol is suppressed by a steroid 5α-reductase inhibitor. Mol Cell Endocrinol.

[23] Chung JS, Zmora N, Katayama H, Tsutsui N (2010). Crustacean hyperglycemic hormone (CHH) neuropeptidesfamily: functions, titer, and binding to target tissues. Gen Comp Endocrinol.

[24] Bray W, Lawrence A. Reproduction of *Penaeus* species in captivity. In: FAaL LJ, editor. Marine shrimp culture: Principles and practices. New York: Elsevier; 1992. p. 93–170.

[25] Caillouet CW (2009). Ovarian maturation induced by eyestalk ablation in pink shrimp, *Penaeus duorarum burkenroad1*. Proc Annual Workshop World Mariculture Soc.

[26] Rotllant G, Nguyen TV, Aizen J, Suwansa-ard S and Ventura T. Toward the identification of female gonadstimulating factors in crustaceans. Hydrobiologia. 2018;825(1):91–119.

[27] Bliss DE, Welsh JH (1952). The neurosecretory system of brachyuran crustacea. Biol Bull.

[28] Mykles DL, Adams ME, Gade G, Lange AB, Marco HG, Orchard I (2010). Neuropeptide action in insects and crustaceans. Physiol Biochem Zool.

[29] Covi JA, Chang ES, Mykles DL (2012). Neuropeptide signaling mechanisms in crustacean and insect molting glands. Invertebr Reprod Dev.

[30] Abuhagr AM, Maclea KS, Chang ES, Mykles DL (2014). Mechanistic target of rapamycin (mTOR) signaling genes in decapod crustaceans: cloning and tissue expression of mTOR, Akt, Rheb, and p70 S6 kinase in the green crab, *Carcinus maenas*, and blackback land crab, *Gecarcinus lateralis*. Comp Biochem Physiol A Mol Integr Physiol.

[31] Das S, Pitts NL, Mudron MR, Durica DS, Mykles DL (2016). Transcriptome analysis of the molting gland (Y-organ) from the blackback land crab, *Gecarcinus lateralis*. Comp Biochem Physiol Part D Genomics Proteomics.

[32] Das S, Mykles DL (2016). A comparison of resources for the annotation of a de novo assembled transcriptome in the molting gland (Y-Organ) of the blackback land crab, *Gecarcinus lateralis*. Integr Comp Biol.

[33] Shyamal S, Das S, Guruacharya A, Mykles DL, Durica DS (2018). Transcriptomic analysis of crustacean molting gland (Y-organ) regulation via the mTOR signaling pathway. Sci Rep.

[34] Das S, Vraspir L, Zhou W, Durica DS, Mykles DL (2018). Transcriptomic analysis of differentially expressed genes in the molting gland (Y-organ) of the blackback land crab, *Gecarcinus lateralis* , during molt-cycle stage transitions. Comp Biochem Phys D.

[35] Covi JA, Chang ES, Mykles DL (2009). Conserved role of cyclic nucleotides in the regulation of ecdysteroidogenesis by the crustacean molting gland. Comp Biochem Physiol A Mol Integr Physiol.

[36] Zmora N, Sagi A, Zohar Y, Chung JS (2009). Molt-inhibiting hormone stimulates vitellogenesis at advanced ovarian developmental stages in the female blue crab, *Callinectes sapidus 2*: novel specific binding sites in hepatopancreas and cAMP as a second messenger. Saline Systems.

[37] Bockaert J, Claeysen S, Becamel C, Pinloche S, Dumuis A (2002). G protein-coupled receptors: dominant players in cell-cell communication. Int Rev Cytol.

[38] Pierce KL, Premont RT, Lefkowitz RJ (2002). Seven-transmembrane receptors. Nat Rev Mol Cell Biol.

[39] Dong C, Zhang P (2012). A putative G protein-coupled receptor involved in innate immune defense of *Procambarus clarkii* against bacterial infection. Comp Biochem Physiol A Mol Integr Physiol.

[40] Filipek S, Teller DC, Palczewski K, Stenkamp R (2003). The crystallographic model of rhodopsin and its use in studies of other G protein-coupled receptors. Annu Rev Biophys Biomol Struct.

[41] Bockaert J, Pin JP (1999). Molecular tinkering of G protein-coupled receptors: an evolutionary success. EMBO J.

[42] Menzaghi F, Behan D, Chalmers D (2002). Constitutively activated g protein-coupled receptors: a novel approach to cns drug discovery. Curr Drug Target -CNS & Neurol Disord.

[43] Tse MT (2013). G protein-coupled receptors: pioneering Frizzled family receptor structure solved. Nat Rev Drug Discov.

[44] Moriyama EN, Strope PK, Opiyo SO, Chen Z, Jones AM (2006). Mining the *Arabidopsis thaliana* genome for highly-divergent seven transmembrane receptors. Genome Biol.

[45] Bargmann CI (1998). Neurobiology of the *Caenorhabditis elegans* genome. Science.

[46] Adams MD (2000). The genome sequence of *Drosophila melanogaster*. Science.

[47] Nagai C, Mabashi-Asazuma H, Nagasawa H, Nagata S (2014). Identification and characterization of receptors for ion transport peptide (ITP) and ITP-like (ITPL) in the silkworm *Bombyx mori*. J Biol Chem.

[48] Veenstra JA (2015). The power of next-generation sequencing as illustrated by the neuropeptidome of the crayfish *Procambarus clarkii*. Gen Comp Endocrinol.

[49] Buckley SJ, Fitzgibbon QP, Smith GG, Ventura T (2016). In silico prediction of the G-protein coupled receptors expressed during the metamorphic molt of *Sagmariasus verreauxi* (Crustacea: Decapoda) by mining transcriptomic data: RNA-seq to repertoire. Gen Comp Endocrinol.

[50] Veenstra JA (2016). Similarities between decapod and insect neuropeptidomes. PeerJ.

[51] Toullec JY, Corre E, Mandon P, Gonzalez-Aravena M, Ollivaux C, Lee CY (2017). Characterization of the neuropeptidome of a southern ocean decapod, the antarctic shrimp *Chorismus antarcticus*: Focusing on a new decapod ITP-like peptide belonging to the CHH peptide family. Gen Comp Endocrinol.

[52] Dickinson PS, Stemmler EA, Barton EE, Cashman CR, Gardner NP, Rus S, Brennan HR, McClintock TS, Christie AE (2009). Molecular, mass spectral, and physiological analyses of orcokinins and orcokinin precursor-related peptides in the lobster *Homarus americanus* and the crayfish *Procambarus clarkii*. Peptides.

[53] Hansen KK, Hauser F, Williamson M, Weber SB, Grimmelikhuijzen CJ (2011). The *Drosophila* genes CG14593 and CG30106 code for G-protein-coupled receptors specifically activated by the neuropeptides CCHamide-1 and CCHamide-2. Biochem Biophys Res Commun.

[54] Laufer H, Biggers WJ (2001). Unifying concepts learned from methyl farnesoate for invertebrate reproduction and post-embryonic development. Am Zool.

[55] Daimon T, Uchibori M, Nakao H, Sezutsu H, Shinoda T (2015). Knockout silkworms reveal a dispensable role for juvenile hormones in holometabolous life cycle. Proc Natl Acad Sci U S A.

[56] Zandawala M, Orchard I (2015). Identification and functional characterization of FGLamide-related allatostatin receptor in *Rhodnius prolixus*. Insect Biochem Mol Biol.

[57] Lorenz MW, Kellner R, Hoffmann KH (1995). A family of neuropeptides that inhibit juvenile hormone biosynthesis in the cricket, *Gryllus bimaculatus*. J Biol Chem.

[58] Wang J, Meyering-Vos M, Hoffmann KH (2004). Cloning and tissue-specific localization of cricket-type allatostatins from *Gryllus bimaculatu*s. Mol Cell Endocrinol.

[59] Stay B, Tobe SS (2007). The role of allatostatins in juvenile hormone synthesis in insects and crustaceans. Annu Rev Entomol.

[60] Duve H, Johnsen AH, Maestro J-L, Scott AG, Jaros PP, Thorpe A (1997). Isolation and identification of multiple neuropeptides of the allatostatin superfamily in the shore crab *Carcinus Maenas*. Eur J Biochem.

[61] Duve H, Johnsen AH, Scott AG, Thorpe A (2002). Allatostatins of the tiger prawn, *Penaeus monodon* (Crustacea: Penaeidea). Peptides.

[62] Huybrechts J, Nusbaum MP, Bosch LV, Baggerman G, Loof AD, Schoofs L (2003). Neuropeptidomic analysis of the brain and thoracic ganglion from the Jonah crab, *Cancer borealis*. Biochem Bioph Res Co.

[63] Hentze JL, Carlsson MA, Kondo S, Nassel DR, Rewitz KF (2015). The neuropeptide allatostatin a regulates metabolism and feeding decisions in *Drosophila*. Sci Rep.

[64] Nassel DR, Winther AM (2010). *Drosophila* neuropeptides in regulation of physiology and behavior. Prog Neurobiol.

[65] Vandersmissen HP, Nachman RJ, Broeck JV (2013). B-type allatostatins and sex peptides.

[66] Conzelmann M, Williams EA, Tunaru S, Randel N, Shahidi R, Asadulina A, Berger J, Offermanns S, Jekely G (2013). Conserved MIP receptor-ligand pair regulates *Platynereis* larval settlement. Proc Natl Acad Sci U S A.

[67] Kramer SJ, Toschi A, Miller CA, Kataoka H, Quistad GB, Li JP, Carney RL, Schooley DA (1991). Identification of an allatostatin from the tobacco hornworm *Manduca sexta*. Proc Natl Acad Sci U S A.

[68] Veenstra JA, Agricola HJ, Sellami A (2008). Regulatory peptides in fruit fly midgut. Cell Tissue Res.

[69] Li B, Predel R, Neupert S, Hauser F, Tanaka Y, Cazzamali G, Williamson M, Arakane Y, Verleyen P, Schoofs L (2008). Genomics, transcriptomics, and peptidomics of neuropeptides and protein hormones in the red flour beetle *Tribolium castaneum*. Genome Res.

[70] Li Y, Hernandez-Martinez S, Fernandez F, Mayoral JG, Topalis P, Priestap H, Perez M, Navare A, Noriega FG (2006). Biochemical, molecular, and functional characterization of PISCF-allatostatin, a regulator of juvenile hormone biosynthesis in the mosquito *Aedes aegypti*. J Biol Chem.

[71] Bachtel ND, Hovsepian GA, Nixon DF, Eleftherianos I (2018). Allatostatin C modulates nociception and immunity in *Drosophila*. Sci Rep.

[72] Veenstra JA (1989). Isolation and structure of corazonin, a cardioactive peptide from the American cockroach. FEBS Lett.

[73] Predel R, Neupert S, Russell WK, Scheibner O, Nachman RJ (2007). Corazonin in insects. Peptides.

[74] Ma M, Chen R, Sousa GL, Bors EK, Kwiatkowski MA, Goiney CC, Goy MF, Christie AE, Li L (2008). Mass spectral characterization of peptide transmitters/hormones in the nervous system and neuroendocrine organs of the American lobster *Homarus americanus*. Gen Comp Endocrinol.

[75] Ma M, Bors EK, Dickinson ES, Kwiatkowski MA, Sousa GL, Henry RP, Smith CM, Towle DW, Christie AE, Li L (2009). Characterization of the *Carcinus maenas* neuropeptidome by mass spectrometry and functional genomics. Gen Comp Endocrinol.

[76] Alexander JL, Oliphant A, Wilcockson DC, Audsley N, Down RE, Lafont R, Webster SG (2017). Functional characterization and signaling systems of corazonin and red pigment concentrating hormone in the green shore crab, *Carcinus maenas*. Front Neurosci.

[77] Tawfik AI, Tanaka S, De Loof A, Schoofs L, Baggerman G, Waelkens E, Derua R, Milner Y, Yerushalmi Y, Pener MP (1999). Identification of the gregarization-associated dark-pigmentotropin in locusts through an albino mutant. Proc Natl Acad Sci U S A.

[78] Veenstra JA (2009). Does corazonin signal nutritional stress in insects?. Insect Biochem Mol Biol.

[79] Žitňan D, Kim YJ, Žitňanová I, Roller L, Adams ME (2007). Complex steroid–peptide–receptor cascade controls insect ecdysis. Gen Comp Endocr.

[80] Kim YJ, Spalovska-Valachova I, Cho KH, Zitnanova I, Park Y, Adams ME, Zitnan D (2004). Corazonin receptor signaling in ecdysis initiation. Proc Natl Acad Sci U S A.

[81] Žďárek J, Nachman RJ, Denlinger DL (2000). Parturition hormone in the tsetse Glossina morsitans. J Insect Physiol.

[82] Roller L, Yamanaka N, Watanabe K, Daubnerová I, Žitňan D, Kataoka H, Tanaka Y (2008). The unique evolution of neuropeptide genes in the silkworm *Bombyx mori*. Insect Biochem Molec.

[83] Li S, Torre-Muruzabal T, Sogaard KC, Ren GR, Hauser F, Engelsen SM, Podenphanth MD, Desjardins A, Grimmelikhuijzen CJ (2013). Expression patterns of the *Drosophila* neuropeptide CCHamide-2 and its receptor may suggest hormonal signaling from the gut to the brain. PLoS One.

[84] Stangier J, Hilbich C, Beyreuther K, Keller R (1987). Unusual cardioactive peptide (CCAP) from pericardial organs of the shore crab *Carcinus maenas*. Proc Natl Acad Sci U S A.

[85] Pulver SR, Marder E (2002). Neuromodulatory complement of the pericardial organs in the embryonic lobster, *Homarus americanus*. J Comp Neurol.

[86] Fu Q, Kutz KK, Schmidt JJ, Hsu YW, Messinger DI, Cain SD, de la Iglesia HO, Christie AE, Li L (2005). Hormone complement of the *Cancer productus* sinus gland and pericardial organ: an anatomical and mass spectrometric investigation. J Comp Neurol.

[87] Chung JS, Wilcockson DC, Zmora N, Zohar Y, Dircksen H, Webster SG (2006). Identification and developmental expression of mRNAs encoding crustacean cardioactive peptide (CCAP) in decapod crustaceans. J Exp Biol.

[88] Qiao H, Fu H, Xiong Y, Jiang S, Zhang W, Sun S, Jin S, Gong Y, Wang Y, Shan D (2017). Molecular insights into reproduction regulation of female Oriental River prawns *Macrobrachium nipponense* through comparative transcriptomic analysis. Sci Rep.

[89] da Silva SR, da Silva R, Lange AB (2011). Effects of crustacean cardioactive peptide on the hearts of two Orthopteran insects, and the demonstration of a Frank-Starling-like effect. Gen Comp Endocrinol.

[90] Ewer J, Reynolds S (2002). Neuropeptide control of molting in insects.

[91] Park JH (2003). Targeted ablation of CCAP neuropeptide-containing neurons of *Drosophila* causes specific defects in execution and circadian timing of ecdysis behavior. Development.

[92] Clark AC, del Campo ML, Ewer J (2004). Neuroendocrine control of larval ecdysis behavior in *Drosophila*: complex regulation by partially redundant neuropeptides. J Neurosci.

[93] Chang JC, Yang RB, Adams ME, Lu KH (2009). Receptor guanylyl cyclases in Inka cells targeted by eclosion hormone. Proc Natl Acad Sci U S A.

[94] Kim YJ, Zitnan D, Cho KH, Schooley DA, Mizoguchi A, Adams ME (2006). Central peptidergic ensembles associated with organization of an innate behavior. Proc Natl Acad Sci U S A.

[95] Zitnan D, Kim YJ, Zitnanova I, Roller L, Adams ME (2007). Complex steroid-peptide-receptor cascade controls insect ecdysis. Gen Comp Endocrinol.

[96] Kim YJ, Zitnan D, Galizia CG, Cho KH, Adams ME (2006). A command chemical triggers an innate behavior by sequential activation of multiple peptidergic ensembles. Curr Biol.

[97] Price D, Greenberg M (1977). Structure of a molluscan cardioexcitatory neuropeptide. Science.

[98] Mercier AJ, Friedrich R, Boldt M (2003). Physiological functions of FMRFamide-like peptides (FLPs) in crustaceans. Microsc Res Tech.

[99] Nambu JR, Murphy-Erdosh C, Andrews PC, Feistner GJ, Scheller RH (1988). Isolation and characterization of a *drosophila* neuropeptide gene. Neuron.

[100] Huybrechts J, Bonhomme J, Minoli S, Prunier-Leterme N, Dombrovsky A, Abdel-Latief M, Robichon A, Veenstra JA, Tagu D (2010). Neuropeptide and neurohormone precursors in the pea aphid, Acyrthosiphon pisum. Insect Mol Biol.

[101] Zoephel J, Reiher W, Rexer KH, Kahnt J, Wegener C (2012). Peptidomics of the agriculturally damaging larval stage of the cabbage root fly Delia radicum (Diptera: Anthomyiidae). PLoS One.

[102] Christie AE (2011). Crustacean neuroendocrine systems and their signaling agents. Cell Tissue Res.

[103] Mercier J, Doucet D, Retnakaran A (2007). Molecular physiology of crustacean and insect neuropeptides. J Pestic Sci.

[104] OuYang C, Liang Z, Li L (2015). Mass spectrometric analysis of spatio-temporal dynamics of crustacean neuropeptides. Biochim Biophys Acta.

[105] Duttlinger A, Berry K, Nichols R (2002). The different effects of three *Drosophila melanogaster* dFMRFamide-containing peptides on crop contractions suggest these structurally related peptides do not play redundant functions in gut. Peptides.

[106] Neves CA, Bhering LL, Serrão JE, Gitirana LB (2002). FMRFamide-like midgut endocrine cells during the metamorphosis in Melipona quadrifasciata anthidioides (Hymenoptera, Apidae). Micron.

[107] Yamanaka N, Zitnan D, Kim YJ, Adams ME, Hua YJ, Suzuki Y, Suzuki M, Suzuki A, Satake H, Mizoguchi A (2006). Regulation of insect steroid hormone biosynthesis by innervating peptidergic neurons. Proc Natl Acad Sci U S A.

[108] Yamanaka N, Roller L, Zitnan D, Satake H, Mizoguchi A, Kataoka H, Tanaka Y (2011). Bombyx orcokinins are brain-gut peptides involved in the neuronal regulation of ecdysteroidogenesis. J Comp Neurol.

[109] Van Hiel MB, Vandersmissen HP, Van Loy T, Vanden Broeck J (2012). An evolutionary comparison of leucine-rich repeat containing G protein-coupled receptors reveals a novel LGR subtype. Peptides.

[110] Sudo S, Kuwabara Y, Park JI, Hsu SY, Hsueh AJ (2005). Heterodimeric fly glycoprotein hormone-alpha2 (GPA2) and glycoprotein hormone-beta5 (GPB5) activate fly leucine-rich repeat-containing G protein-coupled receptor-1 (DLGR1) and stimulation of human thyrotropin receptors by chimeric fly GPA2 and human GPB5. Endocrinology.

[111] Luo CW, Dewey EM, Sudo S, Ewer J, Hsu SY, Honegger HW, Hsueh AJ (2005). Bursicon, the insect cuticle-hardening hormone, is a heterodimeric cystine knot protein that activates G protein-coupled receptor LGR2. Proc Natl Acad Sci U S A.

[112] Webster SG, Wilcockson DC (2013). Mrinalini, Sharp JH: Bursicon and neuropeptide cascades during the ecdysis program of the shore crab, *Carcinus maenas*. Gen Comp Endocrinol.

[113] Wilcockson DC, Webster SG (2008). Identification and developmental expression of mRNAs encoding putative insect cuticle hardening hormone, bursicon in the green shore crab *Carcinus maenas*. Gen Comp Endocrinol.

[114] Sathapondecha P, Panyim S, Udomkit A (2015). A novel function of bursicon in stimulation of vitellogenin expression in black tiger shrimp, *Penaeus monodon*. Aquaculture.

[115] Jaszczak JS, Wolpe JB, Bhandari R, Jaszczak RG, Halme A (2016). Growth coordination during *Drosophila melanogaster* imaginal disc regeneration is mediated by signaling through the relaxin receptor Lgr3 in the prothoracic gland. Genetics.

[116] Garelli A, Gontijo AM, Miguela V, Caparros E, Dominguez M (2012). Imaginal discs secrete insulin-like peptide 8 to mediate plasticity of growth and maturation. Science.

[117] Gontijo AM, Garelli A. The biology and evolution of the Dilp8-Lgr3 pathway: A relaxin-like pathway coupling tissue growth and developmental timing control. Mech Dev. 2018;154:44–50. 10.1016/j.mod.2018.04.005 29715504

[118] Jaszczak JS, Wolpe JB, Dao AQ, Halme A (2015). Nitric oxide synthase regulates growth coordination during *Drosophila melanogaster* imaginal disc regeneration. Genetics.

[119] Mykles DL (2001). Interactions between limb regeneration and molting in decapod crustaceans. Am Zool.

[120] Yu X, Chang ES, Mykles DL (2002). Characterization of limb autotomy factor-proecdysis (LAF (pro)), isolated from limb regenerates, that suspends molting in the land crab *Gecarcinus lateralis*. Biol Bull.

[121] Chandler JC, Gandhi NS, Mancera RL, Smith G, Elizur A and Ventura T. Understanding insulin endocrinology in decapod crustacea: molecular modelling characterization of an insulin-binding protein and insulin-like peptides in the eastern spiny lobster, *Sagmariasus verreauxi*. Int J Mol Sci. 2017;18(9):1832 10.3390/ijms18091832 PMC5618481 28832524

[122] Chandler JC, Aizen J, Elizur A, Hollander-Cohen L, Battaglene SC, Ventura T (2015). Discovery of a novel insulin-like peptide and insulin binding proteins in the Eastern rock lobster *Sagmariasus verreauxi*. Gen Comp Endocrinol.

[123] Golubeva MG (2013). Thyrotropin-releasing hormone: structure, synthesis, receptors, and basic effects. Neurochem J.

[124] Thompson DL, Arana Valencia N (2017). Thyrotropin-releasing hormone: a powerful tripeptide with diverse effects in horses. J Equine Vet Sci.

[125] Sun Y (2003). Thyrotropin-releasing hormone receptors -- similarities and differences. J Mol Endocrinol.

[126] Tanaka Y, Suetsugu Y, Yamamoto K, Noda H, Shinoda T (2014). Transcriptome analysis of neuropeptides and G-protein coupled receptors (GPCRs) for neuropeptides in the brown planthopper *Nilaparvata lugens*. Peptides.

[127] Sotnikova TD, Gainetdinov RR (2009). Octopamine and other monoamines in invertebrates.

[128] Lorenzon S, Brezovec S, Ferrero EA (2004). Species-specific effects on hemolymph glucose control by serotonin, dopamine, and L-enkephalin and their inhibitors in *Squilla mantis* and *Astacus leptodactylus* (crustacea). J Exp Zool A Comp Exp Biol.

[129] Zou HS, Juan CC, Chen SC, Wang HY, Lee CY (2003). Dopaminergic regulation of crustacean hyperglycemic hormone and glucose levels in the hemolymph of the crayfish *Procambarus clarkii*. J Exp Zool A Comp Exp Biol.

[130] Komali M, Kalarani V, Venkatrayulu C, Chandra Sekhara Reddy D (2005). Hyperglycaemic effects of 5-hydroxytryptamine and dopamine in the freshwater prawn, *Macrobrachium malcolmsonii*. J Exp Zool A Comp Exp Biol.

[131] Shimada-Niwa Y, Niwa R (2014). Serotonergic neurons respond to nutrients and regulate the timing of steroid hormone biosynthesis in *Drosophila*. Nat Commun.

[132] Ohhara Y, Shimada-Niwa Y, Niwa R, Kayashima Y, Hayashi Y, Akagi K, Ueda H, Yamakawa-Kobayashi K, Kobayashi S (2015). Autocrine regulation of ecdysone synthesis by beta3-octopamine receptor in the prothoracic gland is essential for *Drosophila* metamorphosis. Proc Natl Acad Sci U S A.

[133] Vazquez L, Alpuche J, Maldonado G, Agundis C, Pereyra-Morales A, Zenteno E (2009). Review: Immunity mechanisms in crustaceans. Innate Immun.

[134] Coast GM, Orchard I, Phillips JE, Schooley DA (2002). Insect diuretic and antidiuretic hormones.

[135] Coast GM, Zabrocki J, Nachman RJ (2002). Diuretic and myotropic activities of N-terminal truncated analogs of *Musca domestica* kinin neuropeptide. Peptides.

[136] Coast GM, Webster SG, Schegg KM, Tobe SS, Schooley DA (2001). The *Drosophila melanogaster* homologue of an insect calcitonin-like diuretic peptide stimulates V-ATPase activity in fruit fly *Malpighian tubules*. J Exp Biol.

[137] Alexander J, Oliphant A, Wilcockson DC, Webster SG (2018). Functional identification and characterization of the diuretic hormone 31 (DH31) signaling system in the green shore crab, *Carcinus maenas*. Front Neurosci.

[138] Cabrero P, Radford JC, Broderick KE, Costes L, Veenstra JA, Spana EP, Davies SA, Dow JA (2002). The Dh gene of *Drosophila melanogaster* encodes a diuretic peptide that acts through cyclic AMP. J Exp Biol.

[139] Meelkop E, Marco HG, Janssen T, Temmerman L, Vanhove MP, Schoofs L (2012). A structural and functional comparison of nematode and crustacean PDH-like sequences. Peptides.

[140] Meelkop E, Temmerman L, Janssen T, Suetens N, Beets I, Van Rompay L, Shanmugam N, Husson SJ, Schoofs L (2012). PDF receptor signaling in *Caenorhabditis elegans* modulates locomotion and egg-laying. Mol Cell Endocrinol.

[141] Iga M, Nakaoka T, Suzuki Y, Kataoka H (2014). Pigment dispersing factor regulates ecdysone biosynthesis via *bombyx* neuropeptide G protein coupled receptor-B2 in the prothoracic glands of *Bombyx mori*. PLoS One.

[142] Suwansa-Ard S, Thongbuakaew T, Wang T, Zhao M, Elizur A, Hanna PJ, Sretarugsa P, Cummins SF, Sobhon P (2015). In silico neuropeptidome of female *Macrobrachium rosenbergii* based on transcriptome and peptide mining of eyestalk, central nervous system and ovary. PLoS One.

[143] On JS, Chow BK, Lee LT (2015). Evolution of parathyroid hormone receptor family and their ligands in vertebrate. Front Endocrinol (Lausanne).

[144] Harmar AJ (2001). Family-B G-protein-coupled receptors. Genome Biol.

[145] Krasnoperov V, Lu Y, Buryanovsky L, Neubert TA, Ichtchenko K, Petrenko AG (2002). Post-translational proteolytic processing of the calcium-independent receptor of alpha-latrotoxin (CIRL), a natural chimera of the cell adhesion protein and the G protein-coupled receptor. Role of the G protein-coupled receptor proteolysis site (GPS) motif. J Biol Chem.

[146] Lin Y (1998). Extended Life-Span and Stress Resistance in the *Drosophila* Mutant methuselah. Science.

[147] West AP, Llamas LL, Snow PM, Benzer S, Bjorkman PJ (2001). Crystal structure of the ectodomain of Methuselah, a *Drosophila* G protein-coupled receptor associated with extended lifespan. Proc Natl Acad Sci U S A.

[148] Patel MV, Hallal DA, Jones JW, Bronner DN, Zein R, Caravas J, Husain Z, Friedrich M, Vanberkum MF (2012). Dramatic expansion and developmental expression diversification of the methuselah gene family during recent *Drosophila* evolution. J Exp Zool B Mol Dev Evol.

[149] de Mendoza A, Jones JW, Friedrich M (2016). Methuselah/methuselah-like G protein-coupled receptors constitute an ancient metazoan gene family. Sci Rep.

[150] Fan Y, Sun P, Wang Y, He X, Deng X, Chen X, Zhang G, Chen X, Zhou N (2010). The G protein-coupled receptors in the silkworm, *Bombyx mori*. Insect Biochem Mol Biol.

[151] Bai H, Zhu F, Shah K, Palli SR (2011). Large-scale RNAi screen of G protein-coupled receptors involved in larval growth, molting and metamorphosis in the red flour beetle. BMC Genomics.

[152] Li C, Zhang Y, Yun X, Wang Y, Sang M, Liu X, Hu X, Li B (2014). Methuselah-like genes affect development, stress resistance, lifespan and reproduction in *Tribolium castaneum*. Insect Mol Biol.

[153] Aizen J, Chandler JC, Fitzgibbon QP, Sagi A, Battaglene SC, Elizur A, Ventura T (2016). Production of recombinant insulin-like androgenic gland hormones from three decapod species: in vitro testicular phosphorylation and activation of a newly identified tyrosine kinase receptor from the Eastern spiny lobster, *Sagmariasus verreauxi*. Gen Comp Endocrinol.

[154] Niwa YS, Niwa R (2014). Neural control of steroid hormone biosynthesis during development in the fruit fly *Drosophila melanogaster*. Genes Genet Syst.

[155] Richter K (1993). Further physiological evidence for nervous regulation of the prothoracic gland in the cockroach *periplaneta-americana*. Zoologische Jahrbucher-Abteilung Fur Allgemeine Zoologie Und Physiologie Der Tiere.

[156] Brody T, Cravchik A (2000). *Drosophila melanogaster* G protein–coupled receptors. J Cell Biol.

[157] Krogh A, Larsson B, von Heijne G, Sonnhammer EL (2001). Predicting transmembrane protein topology with a hidden Markov model: application to complete genomes. J Mol Biol.

